# A B cell screen against endogenous retroviruses identifies glycan-reactive IgM that recognize a broad array of enveloped viruses

**DOI:** 10.1126/sciimmunol.add6608

**Published:** 2024-11-08

**Authors:** Yexin Yang, Rebecca S. Treger, Juan Hernandez-Bird, Peiwen Lu, Tianyang Mao, Akiko Iwasaki

**Affiliations:** 1 Department of Immunobiology, Yale University School of Medicine, New Haven, CT 06520, USA; 2 Department of Molecular, Cellular and Developmental Biology, Yale University, New Haven, CT 06520, USA; 3 Howard Hughes Medical Institute, Chevy Chase, MD 20815, USA

## Abstract

Endogenous retroviruses (ERV), comprising a substantial portion of the vertebrate genome, are remnants of ancient genetic invaders. ERV with near-intact coding potential reactivate in B cell-deficient mice. To study how B cells contribute to host anti-ERV immunity, we employed an antigen-baiting strategy to enrich B cells reactive to ERV surface antigens. We identified ERV-reactive B-1 cells expressing germline-encoded natural IgM antibodies in naïve mice, the level of which further increases upon innate immune sensor stimulation. B cell receptor repertoire profiling of ERV-reactive B-1 cells revealed increased usage of *Igh* V_H_ gene that gives rise to glycan-specific antibodies targeting terminal N-acetylglucosamine moieties on ERV glycoproteins, which further engage the complement pathway to mediate anti-ERV responses. These same antibodies also recognize glycoproteins of other enveloped viruses, but not self-proteins. These results reveal an innate antiviral mechanism of germline-encoded antibodies with broad reactivity to enveloped viruses, which constitutes a natural antibody repertoire capable of preventing the emergence of infectious ERV.

## One Sentence Summary

Profiling of ERV-reactive B-1 cells in mice reveals natural antibodies against terminal N-acetylglucosamine on viral envelope proteins.

## INTRODUCTION

After endogenization of proviral sequences into the host germline, retroviruses gain the capacity to be vertically transmitted through integration into host genome. These events are infrequent but have cumulatively resulted in 8–10% occupancy of murine and human genomes by endogenous retroviruses (ERV) (1, 2). The majority of ERV loci are subject to open reading frame (ORF) degeneration, leaving few copies with intact coding capacity (3). ERV belong to long-terminal repeat (LTR) retrotransposons and based on their homology with exogenous counterparts, murine ERV can be divided into three classes: Class I exemplified by Murine Leukemia viruses (MLV), Class II typified by the mouse mammary tumor virus (MMTV), and Class III that clustered with spumaviruses by sequence homology (4). The endogenous MLV are recent acquisitions of the murine genomes and are actively transcribed and translated in common laboratory mouse strains. A complete MLV locus contains *gag*, *pol*, and *env* genes flanked by two long-terminal repeats (LTR) (4). ERV have established a dynamic equilibrium during co-evolution with the host, balancing their genetic transmission while undergoing a constant arms race with the host control machinery. The surveillance and control of ERV emergence by the host are ensured by cell-intrinsic and extrinsic mechanisms. Despite multiple cell-intrinsic mechanisms which function to silence the activities of ERV loci (5, 6) and restrict cellular synthesis of viral products (7, 8), the resurrection of functionally defective ERV loci in certain immunodeficient mouse strains highlights the non-redundant roles of the host immune system. In particular, deficiencies in B cells and Toll-like receptor 7 (TLR7) signaling result in the emergence of infectious ERV from the C57BL/6 genome (9, 10). On the C57BL/6 background, a single ecotropic MLV (eMLV) locus, *Emv2*, has the capacity to generate infectious virions (11). Although germline *Emv2* encodes a defective *pol* gene and an N-tropic capsid that is targeted by host restriction factor Fv1^b^, this locus can recombine with other xenotropic MLV (xMLV) loci, which restores the inherent defects in *Emv2* to give rise to ERV viremia (9). Active transposition and *de novo* integration events of infectious ERV can cause insertional mutagenesis of host genes, LTR-mediated abnormal oncogene expression, and recombination-induced chromosomal aberrations that ultimately lead to the development of lymphoma and morbidity (9, 10).

Neither T cells nor major histocompatibility complex (MHC) class II presentation is required to prevent ERV emergence on the C57/BL/6 background (9), suggesting that control of ERV is mediated through a T cell-independent (TI) B cell response. In addition, spontaneous ERV emergence is not observed in activation-induced cytidine deaminase (AID)-deficient mice or in immune-competent pups born to ERV viremic recombination-activating gene 1 (Rag1)-deficient breeders. This indicates that the fetal immune repertoire and germline-restricted B cell receptors (BCR) are sufficient to confer protection (9). However, it remains unclear how the fetal germline B cell repertoire mediates the blockade of ERV emergence to prevent subsequent damage to the host.

A major class of TI antibodies is the natural antibodies present in naïve mice prior to infection or immunization, which are predominantly produced by the innate-like B-1 cells. The majority of mouse B-1 cells reside in the peritoneal and pleural cavities (12–14). Generated from precursors in the fetal liver, B-1 cells possess inherent reactivity to self-antigens, upon which their BCR are positively selected during fetal and neonatal hematopoiesis, and to shared structures on foreign antigens (15, 16). B-1 cells that are reactive to pathogen-associated antigens mediate a rapid TI response and generate high quantities of Immunoglobulin M (IgM) and IgG3, functioning as the first line of defense against infections (17, 18). Natural antiviral antibodies, including those against closely related retroviruses, are found in the sera of pre-immune mice (19, 20). Where these antibodies arise and how they contribute to host anti-retroviral immunity is unknown.

In this study, we investigate the requirement of natural IgM and downstream effector molecules in the blockade of ERV emergence. We identified B-1 cells as the major source of natural anti-ERV antibodies through the development of a stringent antigen-baiting strategy. We performed single-cell RNA sequencing (scRNA-seq) and immune repertoire profiling of ERV-reactive B-1 cells, and show that glycan-reactive antibodies are capable of recognition of ERV through terminal N-acetylglucosamine (GlcNAc). We demonstrate that elevated secretion of ERV-reactive antibodies was induced by innate immune stimuli, such as lipopolysaccharide (LPS) and resiquimod (R848). Moreover, these antibodies were found to bind to a broad range of viral glycoproteins and enveloped viral surfaces likely through the recognition of a conserved terminal GlcNAc epitope. Collectively, these results reveal a broadly reactive innate recognition mechanism for viral infection by natural antibodies.

## RESULTS

### Natural IgM suppresses ERV reactivation through complement engagement

**Fig. 1.**
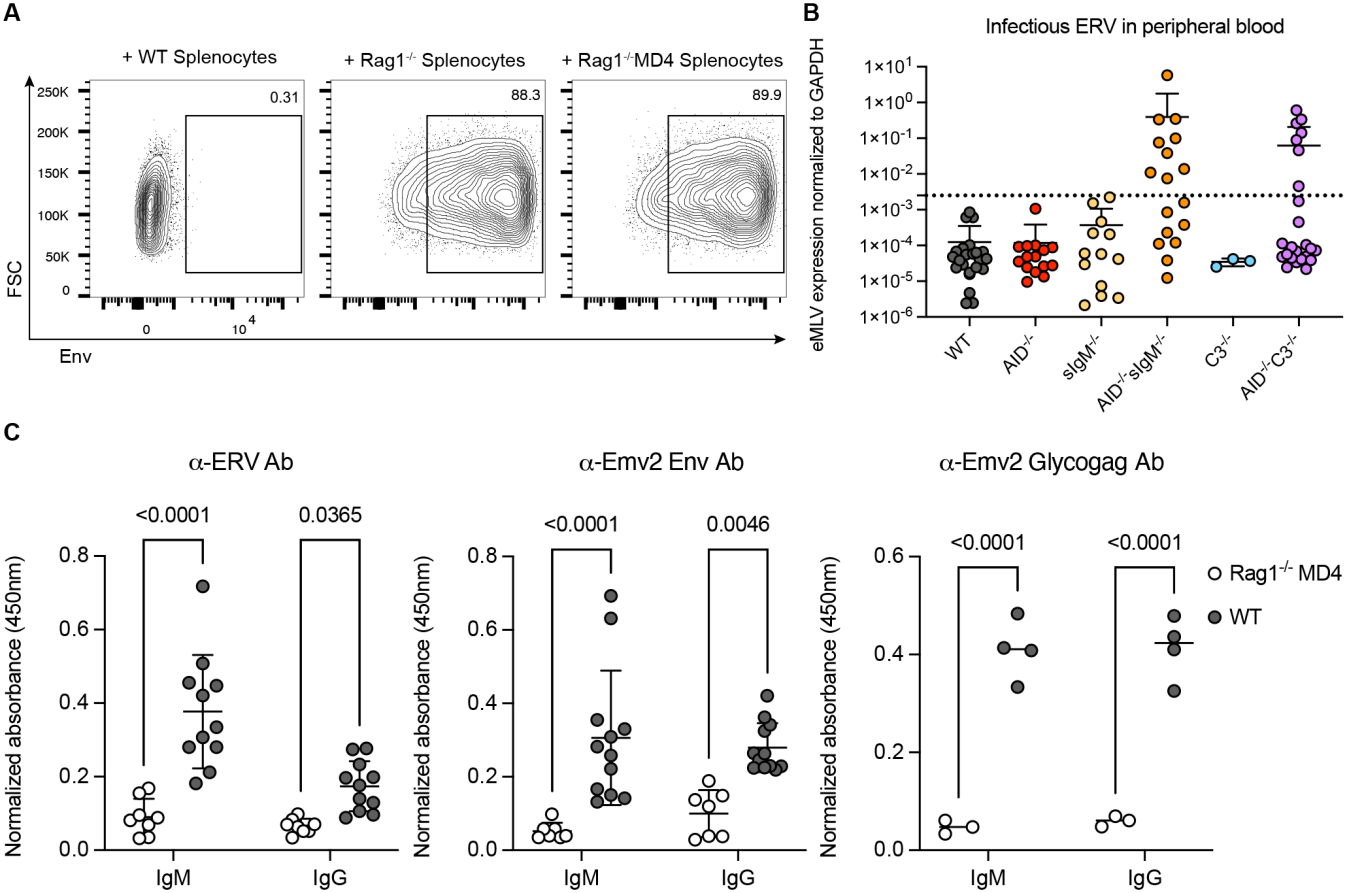
Natural IgM in pre-immune mice suppresses ERV reactivation through complement engagement. (**A**) Representative flow cytometry plots of DFJ8 cells cocultured with indicated splenocytes from WT C57BL/6, *Rag1*^*−/−*^, and *Rag1*^*−/−*^ *MD4 Tg* mice. ERV Env expression by DFJ8 cells was detected by mAb 573. The percentage of Env-positive cells is listed in the upper right of each plot (n=2). (**B**) RT-qPCR of RNA isolated from PBMC of WT or indicated homozygote knockout mice measuring spliced ecotropic Env transcription. Values are normalized to internal GAPDH expression. *AID*^*−/−*^, *sIgM*^*−/−*^ and *C3*^*−/−*^ mice were bred as homozygotes for over 10 generations. *AID*^*−/−*^*sIgM*^*−/−*^ and *AID*^*−/−*^*C3*^*−/−*^ mice were bred as homozygotes for 2–4 generations. Each data point represents an individual mouse. The dotted line represents mean + 3SD of the control group (WT). Values over this cut-off (9/17 of *AID*^*−/−*^*sIgM*^*−/−*^ and 7/24 of *AID*^*−/−*^*C3*^*−/−*^) are considered as high (n=3 to 24). (**C**) ELISA of serum IgM and IgG reactive to ERV particles (left panel), recombinant Env (middle panel), and recombinant Glycogag (right panel) (n=3 to 12). Shown are cumulative or representative results from at least two independent experiments. Data are plotted as mean ± SD, with individual data points representing individual mice; P-values were calculated using two-way ANOVA test with Šídák’s multiple comparisons test.

When the permissive DFJ8 cells, an avian fibroblast cell line that stably expresses the MLV entry receptor (21) were co-cultured with splenocytes isolated from mice with deficiency in B and T cells (*Rag1*^*−/−*^) or with a single invariant BCR to hen egg lysozyme (*Rag1*^*−/−*^*MD4 Tg*), fully infectious ERV were isolated from splenocytes, and infected DFJ8 cells (Figure 1A). Whether ERV control occurs through secreted antibodies or other B cell effector functions is unknown. In addition to the lack of ERV emergence in *AID*^−/−^ (*Aicda*^−/−^) mice (9), ERV emergence was not observed in mice that are unable to secrete IgM (*sIgM*^−/−^) (22), suggesting that class-switched isotypes (e.g., IgG) are independently sufficient to suppress ERV (Figure 1B). To determine whether secreted antibodies are required for inhibiting ERV emergence, we next generated homozygous *AID*^*−/−*^*sIgM*^*−/−*^ mice. Since the emergence of infectious ERV requires several rare recombination events to restore the replication defects in *Emv2* (9), ERV emergence, as indicated by the increase of spliced *Emv2* transcripts in PBMC, was first observed in the second generation (F2) of homozygous knockout breeding (Figure 1B and Figure S1A). The emergence of ERV in *AID*^*−/−*^*sIgM*^*−/−*^ but not in *AID*^−/−^ mice indicates a critical role for secreted antibodies from germline-restricted B cell repertoire in preventing ERV emergence in mice.

Having a multimeric structure, IgM is especially efficient in the initiation of classical complement pathway through C1q deposition on bound IgM (23). We therefore sought to investigate the requirement for complements in ERV suppression by using mice deficient in complement pathway (*C3*^*−/−*^) (24) to generate *AID*^*−/−*^*C3*^*−/−*^ mice. While ERV viremia did not manifest in *C3*^*−/−*^ mice, we observed ERV emergence by the third generation (F3) of *AID*^*−/−*^*C3*^*−/−*^ homozygotes (Figure 1B and Figure S1B), which demonstrated the indispensable role of complement in natural antibody-mediated control of ERV.

We next examined ERV-reactive antibodies in the serum of mice. We detected anti-ERV antibodies in the serum of wild-type (WT) adult C57BL/6 mice prior to immunization (Figure 1C). To rule out non-specific antibody reactivity to antigens derived from the DFJ8 cells in which the ERV particles were produced, we generated a recombinant Emv2 envelope (Env) surface subunit (SU) that retained antigenicity to the rat anti-ERV Env monoclonal antibody (mAb) 83A25 (Figure S2A and S2B), and the glycosylated form of Emv2 Gag protein (Glycogag). Env and Glycogag are the two major extracellular antigenic determinants that ERV-reactive antibodies recognize (25, 26). Similarly, we detected anti-Emv2 Env and anti-Emv2 Glycogag antibodies in the sera of naïve WT mice (Figure 1C). These results collectively show that ERV-reactive natural IgM antibodies are present in the sera of naive mice.

### ERV-reactive B Cells are enriched in the peritoneal B-1 compartment

**Fig. 2.**
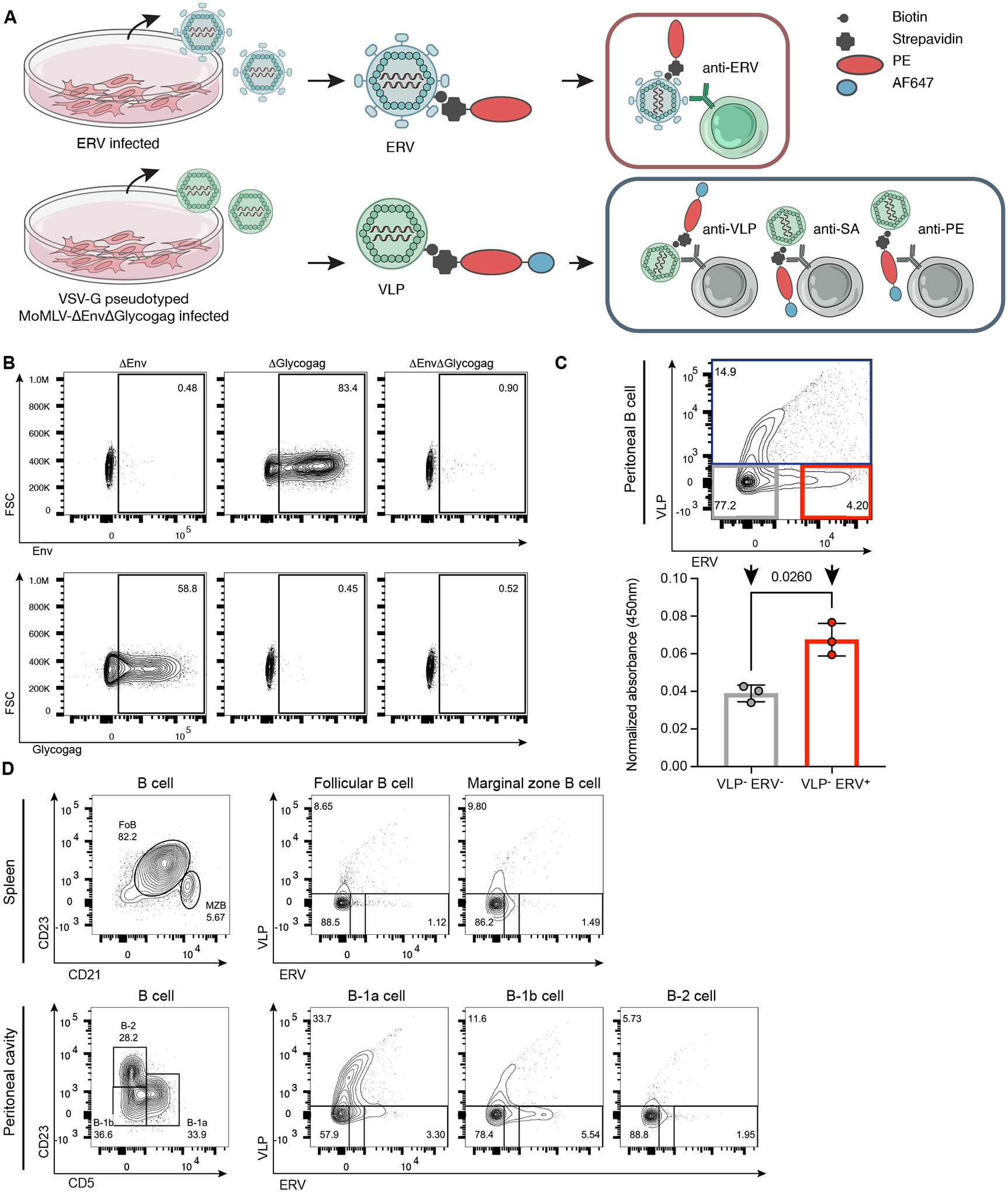
An ERV-baiting strategy enables detection of ERV-reactive B cells in pre-immune splenic and peritoneal compartments. (**A**) Schematic of fluorescence streptavidin-biotin-tagged ERV and VLP baiting strategy. ERV purified from ERV-infected DFJ8 cells were tagged with PE to label ERV-reactive B cells (top). DFJ8 cells infected with VSV-G pseudotyped FMLV-ΔEnvΔGlycogag were cultured for VLP production. Purified VLP were tagged with PE conjugated to AF647 dye to detect non-specific binding (bottom). (**B**) Representative flow cytometry plots of DFJ8 cells transduced with FMLV-ΔEnv, FMLV-ΔGlycogag, and FMLV-ΔEnvΔGlycogag for VLP production. The percentage of Env-positive cells detected by mAb 573 (top row) and of Glycogag-positive cells detected by mAb 34 (bottom row) is listed in the upper right of each plot (n=2). (**C**) A representative flow cytometry plot of peritoneal B cells incubated with tagged VLP and tagged ERV (upper panel). Gated populations include: AF647^−^PE^−^ or VLP^−^ERV^−^ (grey); AF647^+^PE^+^ or VLP^+^ERV^+^ (blue); AF647^−^PE^Hi^ or VLP^−^ERV^+^ (red). The percentage of gated cells is listed within each gate. ELISA of anti-Emv2 Env IgM in the supernatant of FACS-sorted VLP^−^ERV^−^ and VLP^−^ERV^+^ peritoneal B cells at 5 days post cell culture (lower panel). P-values were calculated using two-tailed paired t-test (n=3). (**D**) Representative flow cytometry plots of splenic (top row) and peritoneal (bottom row) B cells from naïve WT C57BL/6 mice (6-week-old) incubated with tagged VLP and tagged ERV. The gating schema for B cell subsets is shown in the first plot of each row. The percentage of VLP^−^ERV^−^, VLP^+^ERV^+^, and VLP^−^ERV^+^ B cells is listed in each respective gate for follicular B cells and marginal zone B cells in the splenic compartment (top row), and for B-1a cells, B-1b cells and B-2 cells in the peritoneal compartment (bottom row) (n=3). Shown are representative results from at least two independent experiments.

To identify the source of ERV-reactive antibodies, we developed an antigen-baiting assay modified from a technique detecting ovalbumin-reactive B cells (27), to directly detect ERV-reactive B cells. ERV particles were biotinylated for tagging with streptavidin R-phycoerythrin (SA-PE) (Figure 2A). To resolve bona fide ERV-reactive B cells against high non-specific background mediated by naïve BCR recognizing biotin, SA, PE, and cell membrane constituent epitopes present on enveloped retroviruses, we generated a control-bait by producing MLV virus-like particles (VLP) lacking the two antigenic determinants Env and Glycogag (Figure 2B). The VLP were biotinylated and tagged with PE-conjugated to Alexa Fluor 647 (AF647) dye (Figure 2A). Thus, any B cells reactive to non-specific epitopes would emit AF647 signal in addition to PE signal in flow cytometry analysis (Figure 2C and Figure S3A). As a validation of the ERV-baiting strategy, fluorescence-activated cell sorting (FACS)-isolated VLP^−^ERV^+^ (AF674^−^PE^Hi^) ERV-reactive B cells secreted increased anti-Emv2 Env IgM compared to VLP^−^ERV^−^ (AF674^−^ PE^−^) non-ERV-reactive B cells (Figure 2C).

The mouse spleen is primarily occupied by bone marrow-derived B-2 cells, with follicular B (FoB) cells located within the follicles and MZB cells located in the marginal zones. Whereas in the peritoneal and pleural cavities, two B-1 cell subsets, termed B-1a and B-1b cells which can be further distinguished based on CD5 expression, are far more abundant than conventional B-2 cells (15). We next used our ERV-baiting strategy to identify ERV-reactive B cells in different B cell compartments from peritoneal lavage and splenic cell suspension of naïve WT C57BL/6 mice. The cells were additionally stained for surface markers to identify FoB cells (CD5^−^CD21^Mid^CD23^Hi^) and MZB cells (CD5^−^CD21^Hi^CD23^Lo^) from splenic B cells (CD3^−^CD19^+^), and to identify B-1a cells (CD5^+^ CD23^−^), B-1b cells (CD5^−^ CD23^−^) and B-2 cells (CD5^−^ CD23^+^) from peritoneal B cells (CD3^−^CD19^+^) (Figure 2D). Among all B cell compartments examined, splenic FoB cells and peritoneal B-2 cells were the least reactive population, having the smallest percentage of cells bound to ERV or VLP. Although B-1a cells exhibited the greatest reactivity to VLP, ERV-reactive B cells were similarly enriched among peritoneal B-1a and B-1b cells, showing a 2–3 fold increase in the percentage of cells exclusively binding to ERV compared to other compartments. Indeed, within the peritoneal subset, the greatest frequency of ERV-reactive B cells (VLP^−^ERV^+^) was detected in the B-1b cell population (Figure 2D and Figure S3B). Using the ERV-baiting strategy, our data indicate that ERV-reactive BCR sequences are predominantly expressed by both peritoneal B-1 subsets, with a larger contribution from B-1b cells compared to the B-1a subset.

### The ERV-reactive peritoneal B-1 BCR repertoire is composed of germline-restricted IgM

**Fig. 3.**
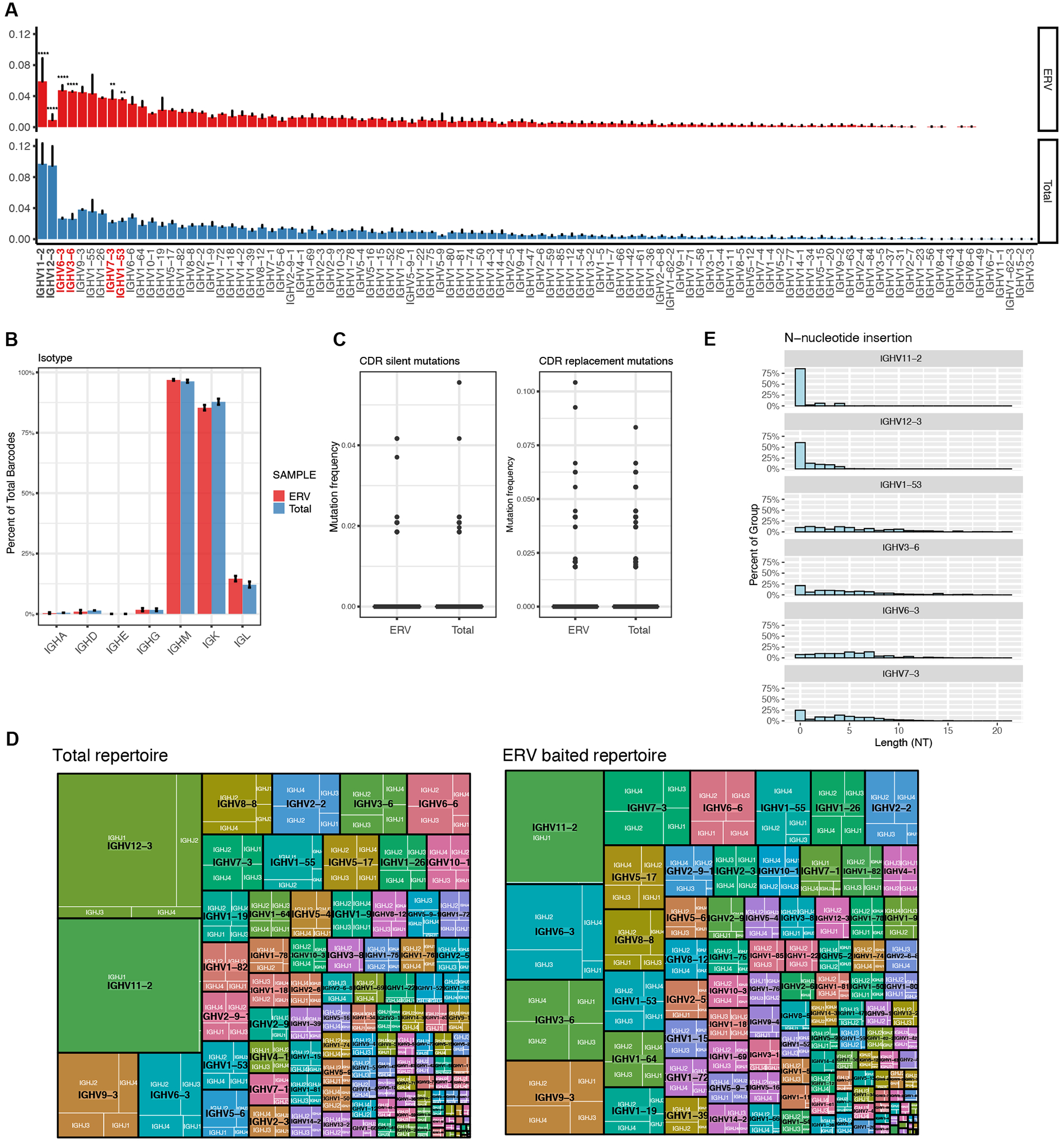
Germline-restricted IgM constituted ERV-reactive peritoneal B-1 BCR repertoire with altered *Igh* V_H_ gene abundance. (**A**) IGHV gene usage profiles are shown as the percentage of IgH sequences encoded by listed individual V_H_ genes for total (blue) and ERV-reactive (red) peritoneal B-1 repertoires. Sequences are from two mice from each group (n=2). ** (p<0.01) and **** (p<0.0001) by unpaired t-test with Holm- Šídák’s multiple comparisons test. (**B**) Isotype profiles are shown as the percentage of sequences encoding the listed class-switched isotypes in total (blue) and ERV-reactive (red) peritoneal B-1 repertoires. Data are pooled from two mice from each group. (**C**) The observed mutation frequencies are categorized into silent mutations (left panel) and replacement mutations (right panel) across CDR sequences in variable regions for total and ERV-reactive peritoneal B-1 repertoires. Sequences are pooled from two mice from each group. (**D**) IgH gene tree-map plots showing V gene and J gene recombination in each BCR for total and ERV-reactive peritoneal B-1 repertoires. Each rectangle in the given tree-map represents a unique V(D)J recombination, and the size of each individual rectangle denotes the relative frequency of the indicated recombination. IGHV (black) and IGHJ (white) gene names are listed in each rectangle. (**E**) Non-templated nucleotide insertion distribution patterns for BCR sequences encoded by each indicated V_H_ gene. Sequences were pooled from all four samples (Total_1, Total_2, ERV_1, ERV_2). Each sample was from an individual mouse.

The antigen-baiting assay identified bona fide ERV-reactive B-1 cells from the total naïve B cells repertoire. To study ERV-reactive B-1 cells, we FACS sorted ERV-reactive (VLP^−^ERV^+^) B-1 cells (CD3^−^CD19^+^CD23^−^) and total peritoneal B-1 cells from naïve WT C57BL/6 mice for paired single-cell RNA sequencing and BCR repertoire sequencing (Figure S4A, Table S1 and S2). We first compared IGHV gene usage between ERV-reactive (3807 cells) and total B-1 cells (9662 cells) to characterize which V_H_ segments are enriched in the ERV-reactive repertoire and to identify putative ERV-reactive BCR. We identified four significantly enriched V_H_ genes: IGHV1–53, IGHV3–6, IGHV6–3, and IGHV7–3 (Figure 3A). The two significantly depleted V_H_ genes in the ERV-reactive repertoire were IGHV11–2 and IGHV12–3, which are known to encode anti-phosphatidylcholine (PtC) BCR and roughly comprise 5–15% of the peritoneal B-1 population (28–30). The vast majority of sequences encoded for the Igμ constant region and thus class-switched antibodies were rarely observed in the ERV-reactive and total repertoires (Figure 3B). Similarly, in the two repertoires, Igκ comprised ~80% of sequenced light chains while Igλ was less frequent (Figure 3B).

We did not observe significant differences in the rate of silent mutation or replacement mutation across predicted complementarity-determining regions (CDR) between ERV-reactive and total BCR repertoires (Figure 3C). Indeed, both repertoires displayed no signs of somatic hypermutation, regardless of V_H_ gene selection. which is consistent with the notion that B-1 cells harbor germline-encoded BCR (31). BCR within the ERV-reactive repertoire exhibited similar average CDR3 length, percentage of basic and acidic residues, and CDR3 hydrophobicity compared to BCR within the total repertoire (Figure S4B). However, when we further examined the diversity of CDR3 by investigating J segments coupling to individual V_H_ genes, we found that significantly distinct from the PtC-reactive IGHV11–2 and IGHV12–3 that predominantly, if not exclusively, recombined with IGHJ1, putative ERV-reactive V_H_ genes did not show selection bias for any of the four J_H_ segments in V(D)J recombination (Figure 3D and Figure S4C). The CDR3 diversity in ERV-reactive BCR is further contributed by non-templated nucleotide insertion at the V(D)J junctions (Figure 3E), which is mediated by terminal deoxynucleotidyl transferase (TdT) induced after birth (32). This observation is indeed in concert with our findings that the largest percentage of ERV-reactive B cells is detected in the B-1b subset, which is replenished by bone-marrow-derived precursors and harbors greater diversity than does the B-1a compartment (15). These findings demonstrate that the ERV-specific BCR repertoire is predominantly comprised of germline-restricted Igμ sequences with diversified CDR3 length and usage of all four J_H_ genes.

### ERV-reactive B-1 clones have unique gene expression patterns

**Fig. 4.**
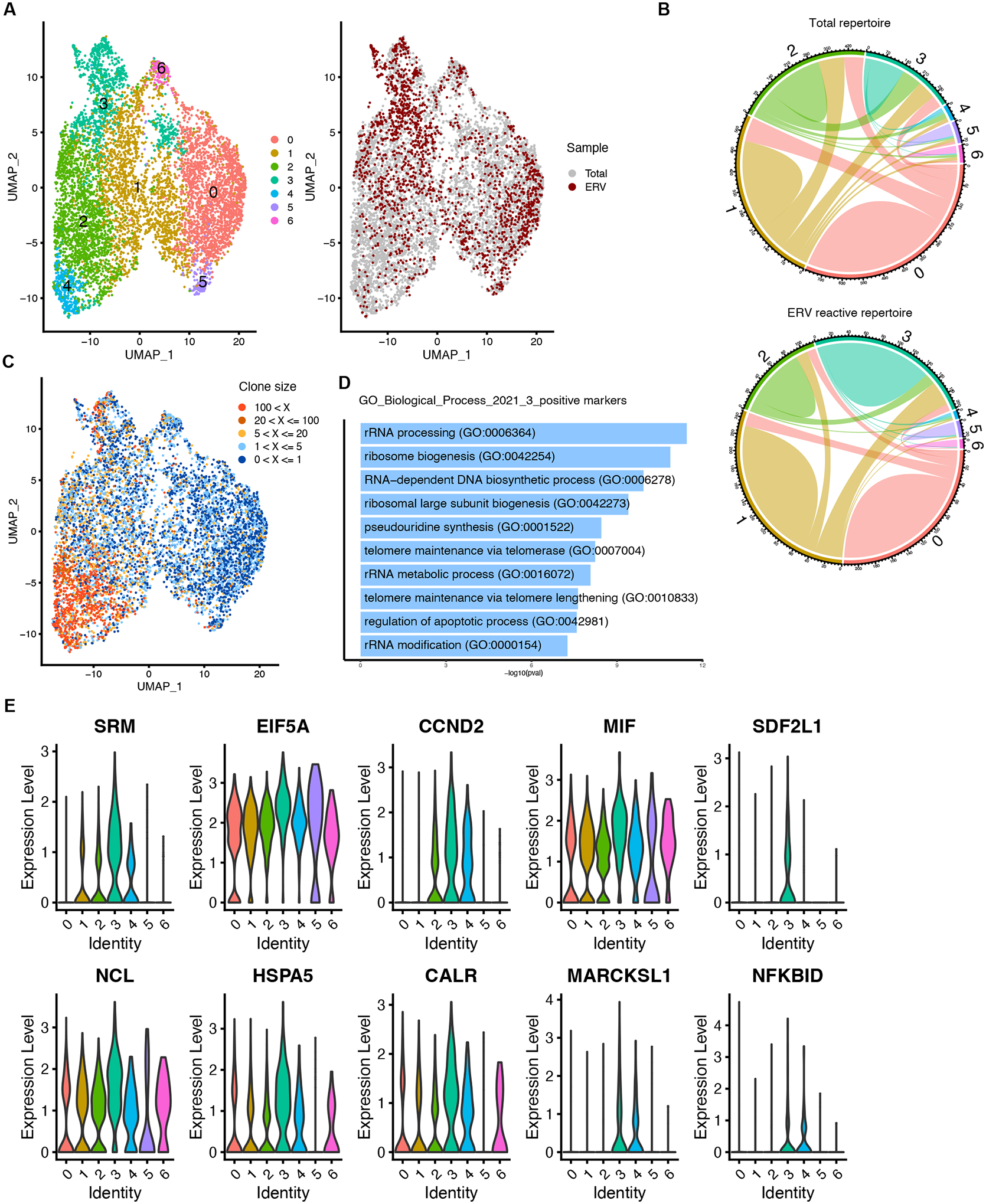
ScRNA-seq identifies a cluster of cells with unique gene expression patterns enriched in ERV-reactive B-1 cells. (**A**) UMAP representation of merged datasets of B-1 cells from the total repertoire and B-1 cells from ERV-reactive repertoire (n=2). Assigned cluster identities are listed (left panel). Cells attributed to total (grey) and ERV-reactive (red) repertoire are overlaid (right panel). (**B**) Chord diagram depicting the clonal relationship of all clusters in total peritoneal B-1 repertoire (top) and ERV-reactive peritoneal B-1 repertoire (bottom). The cord represents the number of clonotypes unique and shared across the multiple clusters. Colors of cord were assigned by clusters. Sequences are pooled from two individual mice from each group. (**C**) UMAP representation of merged datasets of total and ERV-reactive B-1 cells grouped by five levels of total frequencies of unique clones: 1, 0 < X <= 1; 2, 1 < X <= 5; 3, 5 < X <= 20; 4, 20 < X <= 100; 5, 100 < X. Cells that failed to return paired heavy chain and light chain were removed from the dataset when calling clonotypes. (**D**) GO biological process analysis listing pathways that are up-regulated in cluster 3, compared to the other clusters pooled. (**E**) Violin plot of merged datasets of the total and the ERV-reactive B-1 cells depicting transcription level of listed genes. Cluster 3 enriched genes were identified by the Wilcoxon rank sum test (p<0.001 between cluster 3 and other clusters).

We performed single-cell transcriptomic profiling on ERV-reactive and total B-1 cells, for which we also profiled the BCR repertoire. Unbiased hierarchical clustering of 4003 ERV-reactive B-1 cells and 10231 total naïve B-1 cells showed a landscape of six clusters of phenotypically diverse B-1 cells (Figure 4A). Because all sequenced cells are FACS-sorted peritoneal B-1 cells, clear segregation of clusters was not obtained by dimension reduction using Uniform Manifold Approximation and Projection (UMAP). ERV-reactive cells are largely categorized into cluster 0, cluster 1, and cluster 3 (Figure 4A). The percentages of cells assigned to cluster 3 significantly increased in ERV-reactive repertoire compared to the total naïve B-1 repertoire (Figure 4B). Clonal lineage analysis revealed enrichment of large-frequency clones in cluster 2 and cluster 3 (Figure 4C), suggesting the expansion of such clones or large frequencies of convergent recombination. Notably, the ERV-baiting assay increased the percentage of clones in cluster 3 that did not share clonal relationships with other clusters (Figure 4B), suggesting that ERV-reactive B cells are enriched in a clonally and phenotypically distinct population. To avoid the impact of highly expressed Ig genes associated with B cells in the clustering analysis based on the highly variable features, we removed gene transcripts that mapped to the Ig locus. Therefore, the clustering of B cells would not be driven by Ig gene annotation. However, when we integrated gene expression profiles with BCR sequence analysis after clustering, we observed that anti-PtC BCR expressing B cells are strongly segregated into cluster 2 and cluster 4, and to a lesser extent, putative ERV-reactive V_H_ genes were enriched in cluster 3 (Figure S5B). All three clusters exhibit strong B-1 cells features (Figure S5C), as scored according to previously reported B-1 cell signature genes (33), suggesting that both PtC-reactive BCR and ERV-reactive BCR are associated with enhanced B-1 cell transcription programs.

To further characterize different transcriptomic-defined clusters, we examined the differentially expressed genes (DEG) in all clusters and discovered a set of genes highly expressed in cluster 3 (Figure S5D). Gene ontology (GO) analysis on markers of biological processes enriched in cluster 3 suggested anti-apoptotic and pro-survival features of this population (Figure 4D). Specifically, in addition to *Ccnd2* encoding cyclin D2 which is particularly essential for self-renewal of B-1 cells (34), we identified that spermidine synthase (*Srm*) (35, 36), eukaryotic translation initiation factor 5A (*eIF5a*) (37), macrophage migration inhibitory factor (*Mif*) (38) and heat shock protein family A member 5 (*Hspa5*) (39) which have been reported to promote cell proliferation and survival or suppress apoptosis in lymphoid lineages and/or cancer cells, were upregulated in cluster 3 (Figure 4E). Taken together, these findings suggested that ERV-specific B-1 cells possess distinct features consistent with promoting self-renewal and survival.

### IGHV6–3 mAb enhances complement-dependent neutralization of ERV through recognition of terminal GlcNAc

Using the BCR sequences identified by single-cell immune profiling, we generated recombinant monoclonal antibodies to validate these putative ERV-reactive BCRs. For each enriched V_H_ gene including IGHV1–53, IGHV3–6, IGHV6–3, and IGHV7–3, we identified the largest clone within the ERV-reactive repertoire expressing those V_H_ genes and generated a consensus sequence for IGHV1–53, IGHV3–6, IGHV6–3 and IGHV7–3 encoded IgH and their paired IgL based on the largest clones. These IgH and IgL variable regions were cloned into the mouse IgG2c and Igκ backbone vectors respectively to generate monoclonal antibodies (mAb). Except for IGHV3–6 sequences that failed to generate an antibody, we obtained monoclonal antibodies encoded by IGHV1–53, IGHV6–3, and IGHV7–3. The previously reported Lancefield group A carbohydrate (GAC)-reactive antibodies critical in protection against *Streptococcus pyogenes* infection, are also encoded by an IGHV6–3 rearranged BCR (40). We observed that the variable region of mAb HGAC39 (Cγ3) and HGAC78 (Cμ) which are generated from GAC-reactive hybridomas, shared ~87% amino acids homology with the IGHV6–3 ERV-reactive BCR we identified (Figure S6B) (41, 42).

**Fig. 5.**
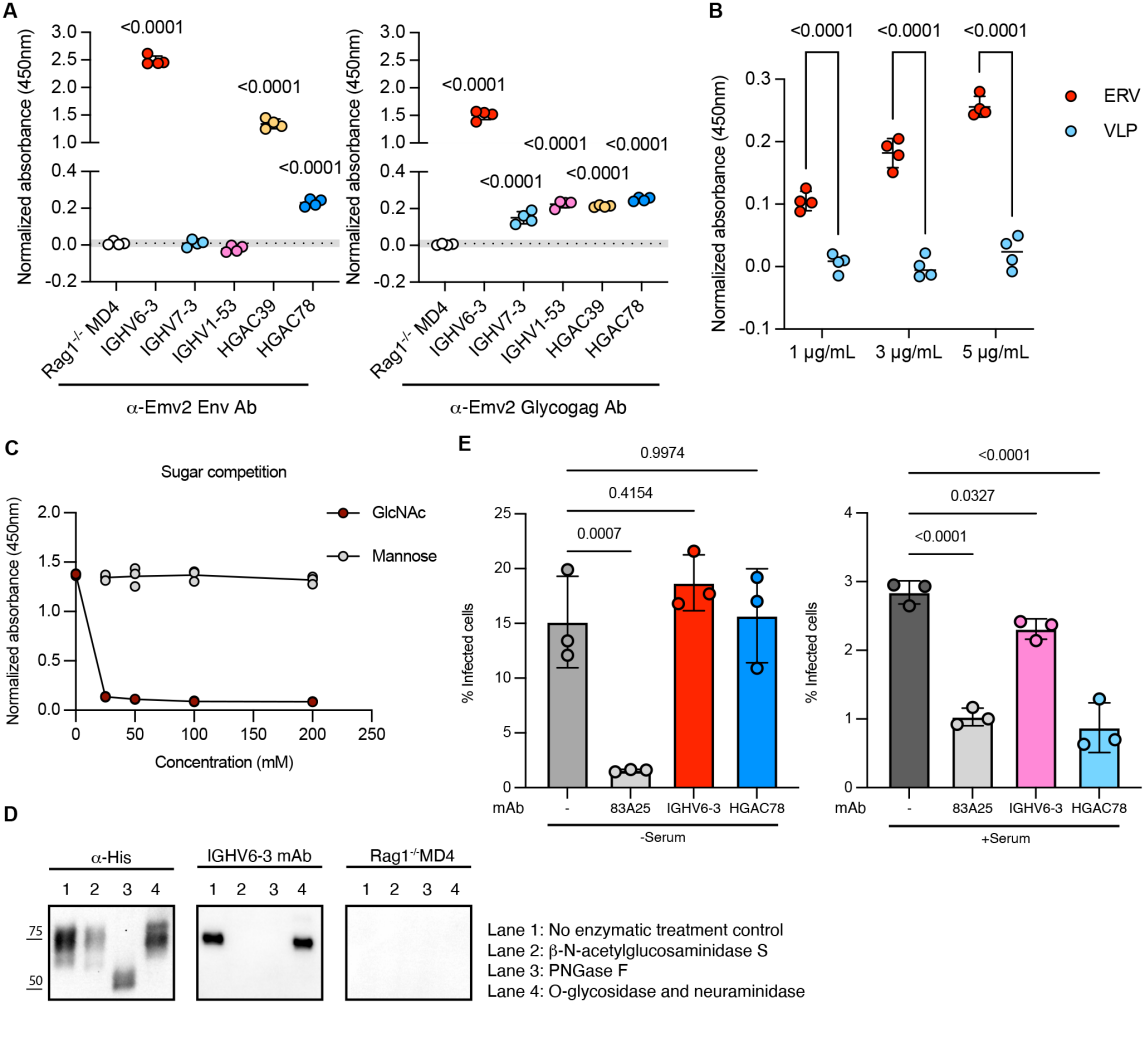
Monoclonal antibody encoded by IGHV6–3 enhances complement-dependent neutralization of ERV through recognition of terminal GlcNAc. (**A**) ELISA of purified recombinant monoclonal antibodies measuring reactivities to ERV Env (left panel) and Glycogag (right panel). Sera from *Rag1*^*−/−*^ *MD4 Tg* mice were used as the negative control. Data are plotted as mean ± SD, with individual data points representing technical replicates. The dotted line and grey filled area represent mean ± 2SD of the negative control. P-values were calculated using one-way ANOVA with Šídák’s multiple comparisons test to the mean value of the negative control (n=4). (**B**) ELISA of IGHV6–3 mAb measuring reactivities to ERV and VLP. Data are plotted as mean ± SD with individual data points representing technical replicates. P-values were calculated using two-way ANOVA with Šídák’s multiple comparisons (n=4). (**C**) Competitive ELISA of IGHV6–3 mAb measuring reactivities to ERV Env after incubation of IGHV6–3 mAb with the indicated concentration of monosaccharides. mAb pre-absorbed with α-methylmannoside were incubated for negative control. Individual data points represent technical replicates (n=3). (**D**) Western blot analysis of denatured Env (Lane 1, control), β-N-Acetylglucosaminidase S-treated Env (Lane 2), PNGase F-treated Env (Lane 3), and O-Glycosidase & Neuraminidase-treated Env (Lane 4). Bands were detected using anti-His Ab, IGHV6–3 mAb, or serum from *Rag1*^*−/−*^ *MD4 Tg* mice (negative control) (n=2). (**E**) Percentage of ERV-infected DFJ8 cells quantified by mAb 573 staining in flow cytometry analysis at 48h post-infection with inhibition mediated by the addition of no mAb (negative control), mAb 83A25 (positive control), IGHV6–3 mAb or HGAC78 mAb without serum (left panel) and with serum (right panel) (n=3). Shown are representative results from at least two independent experiments. Data are plotted as mean ± SD with individual data points representing replicate wells. P-values were calculated using one-way ANOVA with Šídák’s multiple comparisons test to the mean value of the negative control.

We therefore tested the three mAb we generated, in parallel with HGAC39 and HGAC78, for reactivity against ERV antigens. IGHV6–3 mAb reacted strongly to both Env and Glycogag, while HGAC mAb exhibited weaker reactivity to both antigens, and IGHV1–53 mAb and IGHV7–3 mAb only weakly reacted with Glycogag (Figure 5A and Figure S6A). We further verified that IGHV6–3 mAb exclusively recognized Emv2 ERV particles, but not VLP lacking Env and Glycogag antigens (Figure 5B). To investigate which IGHV6–3 mAb domains are required for Env recognition, we constructed domain-swapped antibodies by exchanging each individual CDRs between IGHV6–3 and IGHV7–3 or IGHV1–53 (Figure S6C). While IGHV1–53 mAb did not share sequence homology with IGHV6–3 mAb, IGHV7–3 mAb are ~63% identical to IGHV6–3 mAb across the variable region. Similar to IGHV6–3 clones, IGHV7–3 clones are induced by GAC immunization (40). However, the framework provided by IGHV6–3 appeared to be critical for Env recognition, indicated by the loss of reactivity that occurs when CDRs from IGHV6–3 are grafted onto either IGHV1–53 or IGHV7–3 FWR (Figure S6C).

The epitope for GAC recognition by HGAC mAb is N-acetylglucosamine (GlcNAc) side chains on bacterial polysaccharides (43, 44). Given the cross-reactivity of HGAC mAb to ERV Env, we hypothesized that IGHV6–3 mAb with sequence similarity to HGAC mAb may recognize a similar epitope on ERV. Supporting this hypothesis, IGHV6–3 mAb failed to bind to ERV Env when being saturated with free GlcNAc that can compete away GlcNAc binding site, but not with mannose (Figure 5C). We next explored what types of GlcNAc residues mediate immune recognition by IGHV6–3 mAb. The array of multiple GlcNAc residues on the non-reducing end of GAC O-linked glycans is uniformly expressed in all group A streptococcus serotypes. However, these residues are absent on mammalian cells and are therefore targeted by the host immune system (45, 46). Although structurally distinct from bacterial lipopolysaccharides, the MLV *Env* gene product contains six to eight N-linked oligosaccharides and at least one O-linked glycan, all located on the external gp70 subunit (47). Enzymatic treatments were employed with 1) β-N-acetylglucosaminidase S to exclusively remove terminal non-reducing GlcNAc on ERV Env, 2) PNGase F to remove all N-linked glycans, and 3) a combination of O-glycosidase and neuraminidase to remove O-linked glycans. Interestingly, removing terminal non-reducing GlcNAc is sufficient to abolish IGHV6–3 mAb reactivity, and these terminal GlcNAc are present on N-linked glycans, whereas O-linked glycans were not required (Figure 5D). The cross-reactivity of HGAC mAb to ERV Env was also found to be mediated by terminal GlcNAc (Figure S6D).

After identifying the reactivity of IGHV6–3 mAb for ERV terminal GlcNAc, we sought to validate this in a functional assay. As we identified that natural antibodies in pre-immune mice elicit protection against ERV emergence by engaging complement cascade, we next tested whether IGHV6–3 mAb could inhibit ERV infection of DFJ8 cells in the presence of complement. In the absence of serum, although ERV-specific rat mAb 83A25 efficiently neutralized ERV, neither IGHV6–3 mAb nor HGAC78 mAb reduced ERV infection. However, in the presence of *Rag1*^*−/−*^ *MD4 Tg* serum that contains infectious ERV and complement proteins but no ERV-reactive antibodies, IGHV6–3 (Cγ2c) mAb, and HGAC78 (Cμ) mAb to a greater extent, inhibited ERV infection, which suggests that these antibodies exerted ERV control in a complement-dependent manner, with efficiency correlating with the capacity of each isotype to fix complement (Figure 5E). The reduced infection rate observed in the presence of naïve serum is consistent with earlier studies defining other innate immune mediators in the serum (48–50). Therefore, we compared the infection rates to the baseline of infection when serum is present. Collectively, our results demonstrated that recombinant mAb encoded by IGHV6–3 recognizes ERV through terminal GlcNAc residues and recruits complement to inhibit infection.

### Secretion of ERV-reactive antibodies is driven by innate sensor stimulation

**Fig. 6.**
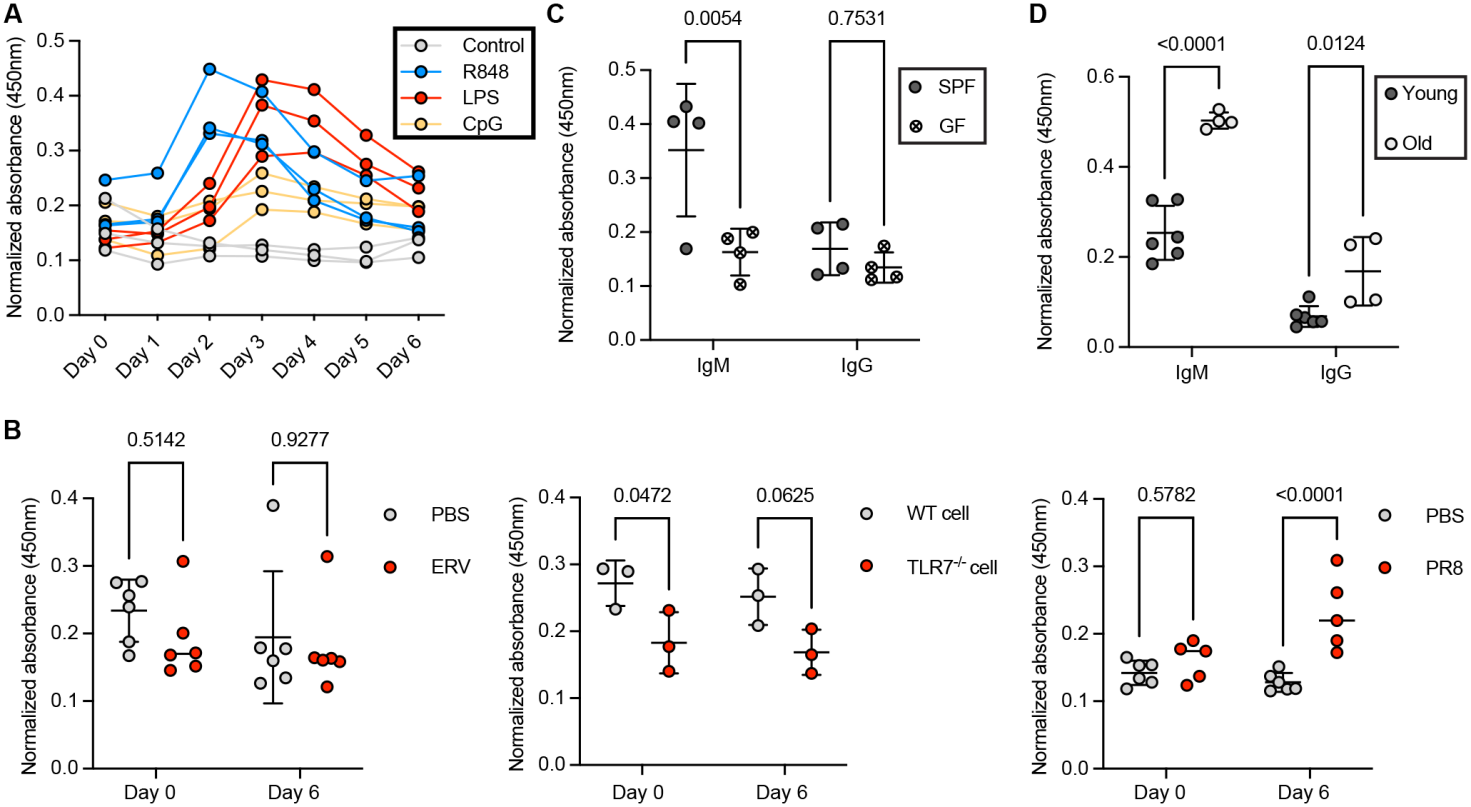
The induction of ERV-reactive antibodies is not antigen-driven but depends on innate sensor stimulation. (**A**) ELISA of Env-reactive IgM detected in the serum of WT mice receiving i.p. injection of PBS (negative control), R848, LPS, or CpG. Timepoints of serum collections from the day of injection are indicated on the x-axis. Lines connecting data points over time represent individual mice (n=3). (**B**) ELISA of Env-reactive IgM detected in the serum of WT mice infected with ERV i.p. (left); WT mice receiving i.p. injection of isolated T cells from TLR7^−/−^ mice (middle); WT mice infected with PR8 i.n. (right). PBS and T cells isolated from WT mice served as negative controls. Each data point represents an individual mouse. P-values were calculated using two-way ANOVA with Šídák’s multiple comparisons (n=3 to 6). (**C**) ELISA of Env-reactive IgM and IgG detected in the serum of WT mice housed in SPF conditions versus a germ-free facility. Each data point represents an individual mouse. P-values were calculated using two-way ANOVA with Šídák’s multiple comparisons (n=4). (**D**) ELISA of Env-reactive IgM and IgG detected in the serum of young (6-week-old) and old (16-week-old) mice (n=4 to 6). Shown are representative results from at least two independent experiments. Each data point represents an individual mouse. P-values were calculated using two-way ANOVA with Šídák’s multiple comparisons.

The ERV viremia in *Tlr7*^*−/−*^ mice demonstrated the requirement of a nucleic acid-sensing pathway for ERV control (10). Moreover, earlier studies have uncovered the role of TLR4, TLR7, and TLR9 in BCR-independent and dependent responses that allow rapid induction of B-1 cell functions, including egress of B-1 cells from the body cavities to the spleen, and differentiation into antibody-secreting cells (51–55). Therefore, to dissect the role of different innate signaling sensing pathways in the induction of anti-ERV natural antibodies, peritoneal B cells from naïve mice were isolated and stimulated *in vitro* with synthetic ligands for different innate sensors. Anti-Emv2 Env IgM was significantly induced upon TLR7 and TLR9 activation with R848 and CpG DNA respectively. This was not observed with Pam3CSK activation of TLR2, Poly(I:C) activation of TLR3, retinoic acid-inducible gene I (RIG-I), and melanoma differentiation-associated protein 5 (MDA5), or stem-loop RNA 14 (SLR14) (56) activation of RIG-I. LPS activation of TLR4 slightly enhanced anti-Emv2 Env IgM secretion *in vitro* (Figure S7A). In naïve mice, intraperitoneal (i.p.) injection of TLR7 and TLR4 agonists rapidly induced serum anti-Emv2 Env IgM as early as day 2 post-injection, while mice that received TLR9 agonist i.p. displayed a minimal response (Figure 6A). We further confirmed that the induction of anti-Emv2 Env IgM secretion by TLR ligands was not restricted to B cells residing in the peritoneal cavity, as it was also observed in isolated splenic B cells, although to a lower level (Figure S7B).

We further observed the insufficiency of cognate antigen encounter for the induction of anti-Emv2 Env IgM, as it was not observed in mice receiving i.p. challenge with infectious ERV particles nor splenic T cells from *Tlr7*^*−/−*^ mice that highly expressed Emv2 ERV Env on the cell surface (Figure 6B). In contrast, WT mice intranasally (i.n.) infected with influenza A strain A/Puerto Rico/8/34 (H1N1; PR8) upregulated anti-Emv2 Env IgM secretion, suggesting that unrelated pathogen challenges that efficiently activate TLR7 through single-stranded RNA (57) without involving cognate antigen recognition are sufficient for induction of anti-Emv2 Env IgM (Figure 6B). Although we were not able to establish acute ERV infection with reactivated ERV particles in mice, we crossed *Tlr7*^+/−^ female breeders with *Tlr7*^−/*y*^ male breeders to obtain TLR7-deficient F1 offspring and monitored de novo ERV emergence in the absence of vertical transmission of ERV from female breeders. As these TLR7-deficient strains were generated on the C57BL/6N background, which possesses elevated levels of non-ecotropic ERV (NEERV) transcripts (5), *Emv2* recombination occurred at an accelerated frequency. We observed ERV emergence in 13/16 F1 offspring (Figure S7C). Compared to TLR7-competent littermates with variable levels of natural ERV-reactive antibodies, none of the TLR7-deficient offspring, whether with or without ERV viremia, produced detectable anti-Emv2 Env IgM. This is in contrast to TLR7-competent mice that sustain ERV viremia (*AID*^*−/−*^*C3*^*−/−*^) and significantly upregulate anti-Emv2 Env IgM, indicating that the TLR7 signaling pathway is required for induction of natural antibodies against ERV (Figure S7C).

Apart from a direct impact on the induction of natural ERV-reactive antibodies, TLR signaling may interplay with BCR signaling that drives clonal expansion of ERV-reactive B-1 cell clones, affecting antibody levels at steady state, as hinted by the near-undetectable level of anti-Emv2 Env IgM in TLR7-deficient mice, regardless of ERV viremia (Figure S7C). Although there are no ERV-free mouse models, a previous study has demonstrated that neonatal exposure to the microbiota drives clonal expansion of IGHV6–3 encoded clones reactive to GAC (40). Indeed, we observed that anti-Emv2 Env IgM was reduced in the serum of germ-free mice at steady state compared to specific pathogen-free (SPF) mice, which is unsurprising given the overlapping epitopes of GAC and ERV that these glycan-specific antibodies recognize (Figure 6C). Both BCR- and TLR-mediated signaling are integral triggers in directing positive selection of B-1 cell clones reactive to microbiome or self-antigens (40, 58). Additionally, we observed that serum anti-Emv2 Env IgM levels significantly increased with age (Figure 6D). Given the short half-life of IgM (59), the observed phenotype reflects the constant shaping of the ERV-reactive clonal repertoire rather than an accumulated effect. Thus, these data underscored the critical function of innate sensing pathways in both inducing ERV-reactive natural antibodies and selecting ERV-reactive clones in the pre-immune repertoire.

### IGHV6–3 mAb targets conserved glycan epitopes on diverse enveloped viruses

**Fig. 7.**
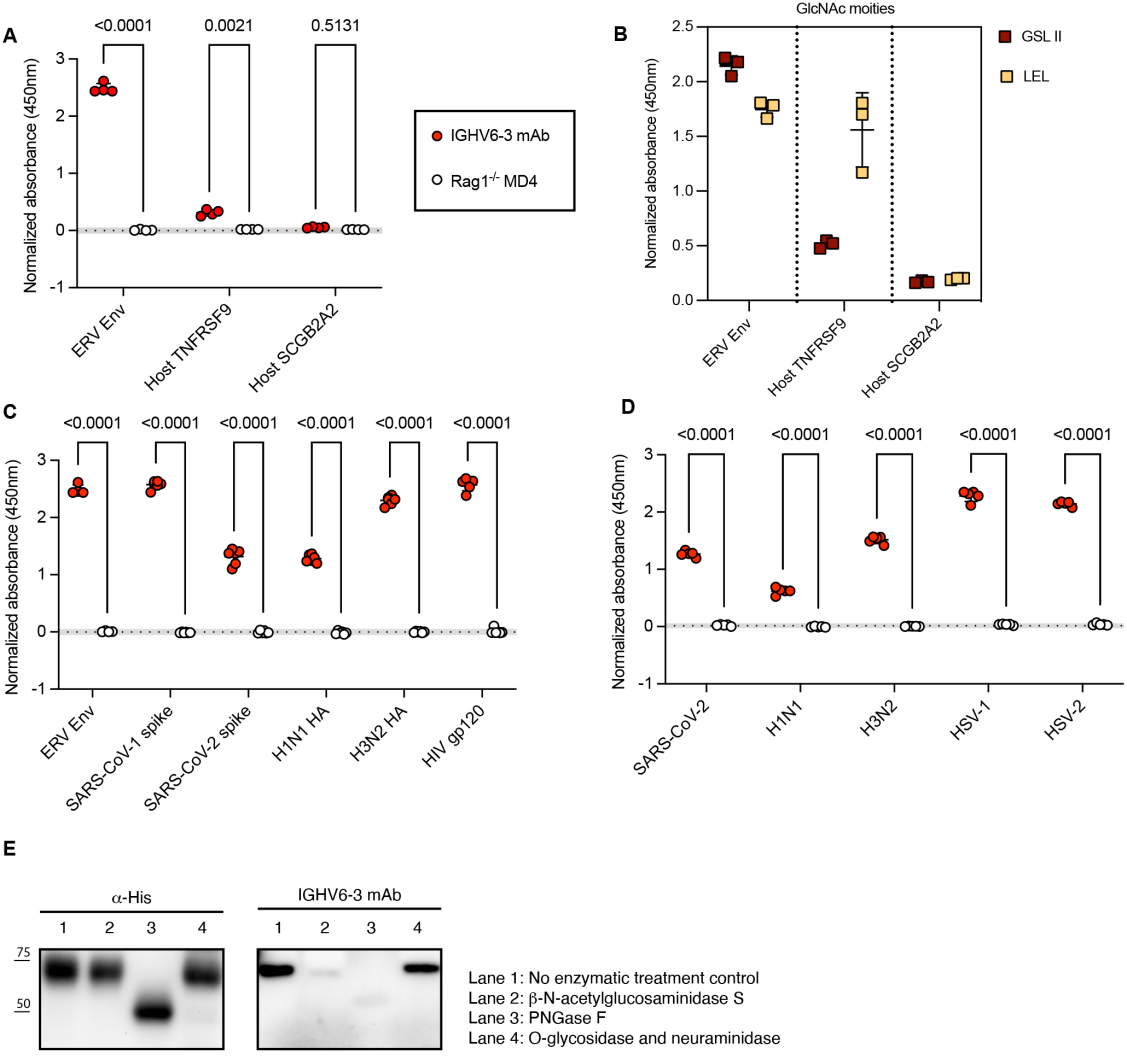
A conserved glycan epitope was targeted by IGHV6–3 mAb for recognition of exogenous viruses. (**A**), (**C**) and (**D**) ELISA of IGHV6–3 mAb measuring reactivity to host proteins (A), viral proteins (C), and intact virus particles (D). Serum from *Rag1*^*−/−*^ *MD4 Tg* mice was incubated as the negative control (n=4 to 6). Data are plotted as mean ± SD, with individual data points representing technical replicates. The dotted line and grey filled area represent the mean ± 2SD of the negative control. P-values were calculated using two-way ANOVA with Šídák’s multiple comparisons. (**B**) ELISA of lectin GSL II and LEL to detect GlcNAc moieties on ERV Env and host proteins. Values are normalized to the negative controls (lectin pre-absorbed with GlcNAc) (n=3). Data are plotted as mean ± SD, with individual data points representing technical replicates. (**E**) Western blot analysis of denatured HA (Lane 1, control), β-N-Acetylglucosaminidase S-treated HA (Lane 2), PNGase F-treated HA (Lane 3), and O-Glycosidase & Neuraminidase-treated HA (Lane 4). Bands were detected using anti-His Ab or IGHV6–3 mAb (n=2). Shown are representative results from at least two independent experiments.

Having revealed the innate signaling-dependent regulation of the ERV-reactive B-1 repertoire, we next asked whether the terminal GlcNAc epitopes recognized by ERV-reactive antibodies could discriminate against “non-self”. Many mammalian proteins are post-translationally modified by N-glycosylation, which is initiated by the attachment of a precursor containing GlcNAc. The termini of N-glycans are further processed by galactosylation, sialylation, and/or fucosylation, except for high mannose-type glycans (60). Therefore, host-derived glycoproteins also contain GlcNAc in the core structure. However, IGHV6–3 mAb did not recognize two host proteins containing predicted N-linked glycosylation sites (only by sequence prediction) (Figure 7A). This is also consistent with our results showing that IGHV6–3 mAb failed to recognize VLP lacking ERV surface antigens and Env lacking terminal GlcNAc (Figure 5B and 5D). Using Griffonia simplicifolia II lectin (GSL-II) that recognizes GlcNAcα1–4Galβ1–4GlcNAc (terminal GlcNAc), and Lycopersicon esculentum lectin (LEL) that recognizes (GlcNAcβ1–4)_1–4_ (GlcNAc in core structures) (61), we were able to detect core GlcNAc on ERV Env and TNFRSF9 while terminal GlcNAc was only detected on ERV Env (Figure 7B).

We further extended our investigation to exogenous viruses considering that N-linked glycosylation of envelope proteins is a universal feature of enveloped viruses, and terminal GlcNAc may be present on other envelope glycoproteins. IGHV6–3 mAb bound to recombinant severe acute respiratory syndrome coronavirus 1 (SARS-CoV-1) spike protein, SARS-CoV-2 spike protein, influenza A H1N1 hemagglutinin (HA), influenza A H3N2 HA, and human immunodeficiency virus (HIV) gp120 (Figure 7C). In addition to purified recombinant envelope proteins, IGHV6–3 mAb also recognized live virus particles of SARS-CoV-2, H1N1, H3N2, herpes simplex virus type 1 (HSV-1), and HSV-2 (Figure 7D). Murine IGHV6–3-encoded mouse-human chimeric mAb (Figure S8A) is also reactive to the bronchoalveolar lavage fluid (BALF) of SARS-CoV-2 infected mice (Figure S8B) and the vaginal wash of HSV-2 infected mice (Figure S8C). To further investigate whether such cross-reactivity relies on conserved GlcNAc epitope, we applied enzymatic treatment to remove terminal GlcNAc, N-linked glycans, and O-linked glycans from H1N1 HA. We observed that terminal GlcNAc on N-linked glycans was indispensable for IGHV6–3 mAb recognition of H1N1 HA (Figure 7E). Indeed, rapid B-1 cell secretion of IgM in response to influenza virus infection has been shown to confer early protection, contributing alongside the conventional B-2 cell-derived humoral response to combat infection (18, 62). Thus, relying on the recognition of a conserved glycan epitope ubiquitously displayed on envelope glycoproteins, ERV-reactive natural antibodies recognize a variety of glycoproteins of enveloped viruses. In addition to IGHV6–3 mAb, the IGHV1–53 mAb exhibited strong binding to the H1N1 HA of influenza A virus (Figure S8D), further suggesting that ERV-reactive BCRs cross-react with other viral envelope proteins.

Finally, we examined whether there are human antibodies with similar specificities as the mouse IGHV6–3. The closest related human IGHV genes to the C57BL/6 IGHV6–3 gene all belong to the IGHV3 family, including IGHV3–15, IGHV3–73, IGHV3–72 and IGHV3–74. Interestingly, the human IGHV3 family genes exhibit broad reactivity to polysaccharides antigens from *Streptococcus pneumoniae*, which was demonstrated by antibodies encoded by unmutated germline V genes (63, 64). A human germline IGHV3–15-encoding mAb was previously reported to recognize SARS-CoV-2 spike. In addition, putative prenatal B-1 cells have been identified in humans. In the putative human B-1 repertoire, IGHV1–8 is significantly enriched (65). We generated two recombinant mAbs encoded by germline human IGHV3–15 and IGHV1–8. The heavy and light chains variable sequences of the two mAbs are encoded by putative human B-1 BCR (65). The human IGHV3–15 mAb exhibited reactivity to SARS-CoV-1 spike, SARS-CoV-2 spike, and Emv2 Env (Figure S8E). The germline-encoded motif for glycan recognition in the CDR1 of the human IGHV3 family genes is conserved in murine IGHV6–3 (66), suggesting the human counterpart natural antibodies with germline V genes with glycan reactivity and their role in pathogen recognition.

## DISCUSSION

Endogenous retroviral proteins are transcribed and expressed by the host cells at low levels at steady state, due to incomplete silencing of ERV by epigenetic mechanisms. Furthermore, certain immunodeficiencies can render the host susceptible to ERV reactivation in mice (9, 10), underscoring the indispensable role of the immune system in blocking ERV emergence. While previous studies identified that B cells and nucleic acid sensing by TLR7 are indispensable for ERV control, it is not known which B cell subsets and downstream effector molecules are required. In this study, we employed fluorescence-tagged ERV particles and demonstrated that naïve peritoneal B-1 subsets express ERV-reactive BCRs. This finding is consistent with an earlier observation that anti-MLV antibodies are present in the sera of naïve animals (19, 67). In contrast to conventional B-2 BCRs that are fine-tuned by antigen-directed affinity maturation, B-1 BCRs are largely germline-restricted and considered to be evolutionarily selected for recognition of conserved epitopes present on invading pathogens or dying cells (31). It is well-established that the pre-immune repertoire is constantly being shaped by self- and environmental antigens and exhibits expansion of certain clones and decline of clonal plasticity (40, 58, 68). The timing of dramatic skewing of the pre-immune repertoire coincides with the first exposure to microbiota at birth, and recolonizing germ-free mice with normal microbiota reconstitutes IGHV6–3 clones (40). The self-antigen responsible for the positive selection of ERV-reactive clones during fetal development in the absence of microbiota is nonetheless unclear. Moreover, similarities of the VH1 family gene usage in the B-1 repertoire developed in germ-free mice and SPF mice suggest that unknown self-antigens may help to regulate B-1 cell ontogeny. ERV envelope and glycosylated gag proteins are present on the surface of host cells and could serve as the self-antigen for ERV-reactive B-1 cell selection. Given the long co-evolution with ERV in the genome and the dynamic regulation of ERV transcripts during embryonic development (69, 70), ERV-derived antigens may contribute to fetal B-1 cell selection by providing positive selection cues. From an evolutionary perspective, such a model would explain how stochastic cell-intrinsic loss of ERV repression by fetal cells could be suppressed by the immune system. Notably, memory B cells secreting only IgM, and no IgA, with cross-reactivity to both microbiota-derived antigens and ERV were identified in germ-free mice in the absence of immunization (71), further supporting the hypothesis that ERV, in conjunction with the microbiota, contributes to the development of pre-immune B cell repertoire.

There is a developing model that B-1 cells are heterogeneous in their functionality, both maintaining the natural antibody pool and responding to infections (72). Although B-1 cells located in the spleen and body cavity are not completely segregated due to the systemic circulation (73), B-1 cells in the peritoneal and pleural cavities are specialized to be the rapid “responders” to infection, thereby contributing to induced natural immunity (74). Redistribution of such responder B-1 cells to the spleen, mesenteric lymph node, or mediastinal lymph node, where they differentiate into antibody-secreting cells, was seen in mice receiving either intraperitoneal or intranasal challenge (75–77). We demonstrated that anti-Emv2 Env IgM was inducible upon direct TLR stimulation and by unrelated viral challenge, and the time course for induction of these antibodies preceded the initiation of the B-2 response. ERV transcription is up-regulated in the small intestine by microbial stimulation (78), and depletion of commensal microbiota can prevent ERV emergence (9). Collectively, these data suggest that early ERV production in mice may originate from the gastrointestinal (GI) tract. Given the cross-reactivity of natural IgM and gut IgA to commensal bacterial antigens and ERV, the selection of ERV-reactive clones by microbial antigens, and the correlation of microbial stimulation with ERV transcription, B-1 cells that reside in the peritoneal cavity may be specifically suited to the surveillance and suppression of emerging ERV.

Using our ERV-baiting strategy, we were able to directly profile the ERV-reactive BCR repertoire in naïve mice. Using genetic tools, we demonstrated the requirement for secreted antibodies to prevent ERV emergence. Further, ERV-reactive BCR sequences were identified in this study. Usage of IGHV1–53, IGHV3–6, IGHV6–3, and IGHV7–3 V_H_ genes were enriched in the ERV-reactive repertoire. Consistent with known B-1 BCR features, antibodies encoded by these V_H_ genes appeared to be broadly reactive. Although we did not obtain an IGHV3–6 mAb, two out of the three mAbs generated from IGHV3–6-expressing B-1 cells by other groups were reactive to microbiota (58). The IGHV1–53 mAb we generated were reactive to influenza A virus and murine MZB cells expressing IGHV1–53 react with HIV gp120 (79). IGHV6–3- and IGHV7–3-expressing B-1b cells were reactive to bacterial GAC (40). We further demonstrated that the cross-reactivity of IGHV6–3 mAb stemmed from the capacity of the germline IGHV6–3 framework to recognize exposed terminal GlcNAc epitopes displayed on multiple pathogens, including Group A streptococcus (GAS) and enveloped viruses. Although the IGHV6–3 mAb we identified is ~87% identical to HGAC mAb developed from mice immunized with a streptococcal vaccine, it expresses a distinct CDR3 (Figure S6B). Differences in CDR3 sequences may underlie their preference in specificities to viral-derived N-linked GlcNAc or bacterial-derived clustered O-linked GlcNAc, and further suggest shaping of the pre-immune repertoire upon antigen challenge. The involvement of these IGHV genes in the convergent host humoral immune response against *Plasmodium falciparum* (80), Ebola virus (81), and SARS-CoV-2 (82) were also reported in earlier studies. Given that antibodies encoded by germline IGHV3 family genes were also induced against porcine hepatocytes in human xenotransplantations (83), it is therefore intriguing to identify the epitopes of these germline broadly reactive antibodies and to dissect their role in the recognition of foreign antigens from both pathogens and mammalian sources.

In contrast to bacterial products that are generated using distinct glycosylation systems in prokaryotic organisms, viruses utilize host translation and glycosylation machinery to generate their glycoproteins. It is well established that most of the viral envelope proteins are intensively glycosylated with dense glycosylation sites, which on one hand contributed to immune evasion, a so-called “glycan shield” (84). On the other hand, premature glycosylation could give rise to neoepitopes that permit immune recognition, of which high-mannose type glycan is a well-studied example (85, 86). Oligomannose-type glycans intensively decorate HIV envelope trimers, while on host proteins complex-type glycans are found more abundantly. It is proposed that structural constraint in the envelope closed confirmation may prevent endoplasmic reticulum (ER) α-mannosidase I from accessing the premature glycans on gp120, resulting in stalling of the glycan processing (87, 88). High mannose glycans were also found on influenza virus and SARS-CoV-2 (89, 90), suggesting that a shared mechanism may underlie the generation of immunogenic glycan epitopes across different viral strains.

Limitations of this study include the lack of identification of the exact molecular glycan targets recognized by the ERV-reactive mAbs. We also did not solve the structure of mAbs bound to the target viral envelope proteins. Additionally, further work is needed to optimize and enhance the antiviral activities of the mAbs. Overall, this study exemplifies the role of innate-like antiviral immunity mediated by ERV-reactive natural antibodies potentially contributing to protection against ERV emergence and suggests a role in defense against exogenous enveloped viral infections.

Future study is required to identify the biosynthetic pathways leading to the exposure of terminal GlcNAc on viral glycoproteins, but not host glycoproteins. Notably, besides bacterial- and viral-derived terminal GlcNAc epitopes, such glycan epitopes have been identified on malignant cells (91–93) and in autoimmune disease (94). In fact, proteins with exposed terminal GlcNAc are targeted by mannose-binding lectin (MBL) for removal in the liver, leading to short-term circulatory survival (95, 96).

In summary we identified terminal GlcNAc as a conserved epitope targeted by natural antibodies to discriminate “non-self”. As natural IgM recognizes glycan modifications that did not necessarily occur at the interface between the viral entry protein and its host receptor, they are not sufficient to neutralize viruses. However, as demonstrated in this study, natural antibodies do exert a critical anti-ERV function to protect the host by engaging complement.

## MATERIALS AND METHODS

### Study design

The objective of the current study was to investigate the mechanism by which the naïve germline repertoire of natural antibodies mediate surveillance and prevention of ERV reactivation in C57BL/6 mice. ERV reactivation was detected by RT-PCR and coculture assay. An antigen-bait BCR enrichment assay was developed to identify ERV-reactive B-1 cells in unimmunized mice. BCR repertoire and transcriptome profiling were conducted to reveal IGHV gene usage and gene expression profiles in ERV-reactive B-1 repertoire. ERV reactivities of serum and mAb cloned from the B-1 cells were measured by ELISA. Western blotting and competitive ELISA were performed to identify the specificity of IGHV6–3-encoded mAb derived from one of the largest ERV-reactive B-1 clones. Mice used in the control group and experimental group were age- and sex-matched and assigned randomly to the groups at the beginning of the experiments. Investigators were not blinded because no subjective measurements were performed. The number of replications and statistical analysis performed in each experiment were indicated in the section Quantification and statistical analysis.

### Mice

WT C57BL/6 mice (Cat# C57BL/6NCrl) were obtained from Charles Rivers and bred in-house. *Rag1*^*−/−*^ mice (Cat# 002216; RRID: IMSR_JAX:002216) and *MD4 Tg* mice (Cat# 002595; RRID: IMSR_JAX:002595) were obtained from The Jackson Laboratory and bred in-house. *Rag1*^*−/−*^ mice and *MD4 Tg* mice were backcrossed ten times to C57BL/6 inbred mice. *Tlr7*^*−/−*^ mice (97,104) were bred-in house. *sIgM*^*−/−*^ mice (22) were kindly gifted by Nicole Baumgarth. *AID*^*−/−*^ mice (98,105) were kindly gifted by David Schatz. *C3*^*−/−*^ mice (24) were kindly gifted by Jeanne Hendrickson. *AID*^*−/−*^ mice and *C3*^*−/−*^ mice were backcrossed more than eight times to C57BL/6 inbred mice. B6.Cg-Tg(K18-ACE2)2Prlmn/J (K18-hACE2) mice (stock no. 034860) were obtained from the Jackson Laboratory and subsequently bred in-house. All animal protocols were approved by the Institutional Animal Care and Use Committee (IACUC) at Yale University. Mice were housed in specific pathogen-free facilities and care was provided in accordance with Yale University IACUC guidelines (protocol #10365). Germ-free WT C57BL/6 mice were generously shared by Noah Palm and were hosed in the gnotobiotic facility at Yale University. Mice used in each experiment were strictly age- and sex-matched. Both male and female mice were used in this study. Sex variation was determined to not affect anti-ERV antibody levels at steady state in pilot experiments. Age was determined to affect anti-ERV antibody levels at steady state. In Figure 1C and Figure 6D, mice between the ages of 6 and 16 weeks were used. In Figure 6B, 10-week-old mice were used and in all other *in vivo* experiments, mice between the ages of 6 to 8 weeks were used.

### DFJ8 coculture assay

Co-culture assays were performed as previously described (99). DFJ8 cells were pre-seeded at 100,000 cells/mL in a 12-well-plate one day prior assay with 1 mL Dulbecco’s Modified Eagle Medium (DMEM) per well. Splenocytes were isolated according to the cell isolation method described in this study and added into DFJ8 cell culture with 1.5 mL Roswell Park Memorial Institute (RPMI) medium plus 0.5 mL DMEM. Cells were subcultured to allow replication. On Day 14 post-initial co-culture in 12-well-plate, cells were harvested and stained for mouse CD45 (BioLegend) and for ERV Env by mAb 573 (kindly provided by Leonard Evans, NIH) (100). Percentage of ERV infected cells were quantified in CD45^−^ DFJ8 cells.

### ERV and VLP generation

Single-cell colonies of DFJ8 cells co-cultured with *Tlr7*^*−/−*^ splenocytes that stably express high levels of ERV were cultured for 7 days to amplify ERV particles as described previously (7). Culture supernatants were centrifuged and passed through a 0.45 μm filter to remove DFJ8 cells and cell debris and then concentrated by ultracentrifugation for 23,000 × g over 25% sucrose. The pelleted ERV was resuspended in optiMEM media and stored at −80°C for future analysis. The FMLV-ΔGlycogag vector was kindly gifted by Walther Mothes, Yale University. To generate FMLV-ΔEnvΔGlycogag vector, seamless DNA cloning was performed using In-Fusion Snap Assembly Master Mixes (TAKARA) according to the manufacturer’s manual to delete the Env coding sequence. Fragment 1 was amplified by the FMLV-IF-1F and FMLV-IF-1R primer pair. Fragment 2 was first amplified by the FMLV-IF-2FS and FMLV-IF-2R primer pair, and then amplified with FMLV-IF-2FM and FMLV-IF-2R primers to avoid multiple primer binding sites. Fragment 3 was amplified by the FMLV-IF-3F and FMLV-IF-3R primer pair. All primer sequences are listed in Table S3 and all PCR reactions were conducted using Q5^®^ High-Fidelity 2X Master Mix (NEB). After seamless cloning of all amplified fragments, the joined plasmid was recovered from transformed One Shot^™^ TOP10 E. coli (ThermoFisher) and Sanger sequencing was performed to confirm the sequence across the entire plasmid. 293T cells were transfected with FMLV-ΔEnvΔGlycogag and VSV-G vectors using Lipofectamine^™^ 2000 (Invitrogen) according to the manufacturer’s manual. 24 hours after transduction, the media was replaced. 48 hours after transduction, supernatants containing pseudotyped viruses were harvested and the viruses were concentrated using Retro-X^™^ Concentrator (TAKARA). DFJ8 cells seeded the day before transduction were infected with the concentrated virus in OptiMEM containing 5 μg/mL polybrene. After 2.5 hours, the infected media was removed and replaced with fresh DMEM. Seven days after transduction, DFJ8 cells were harvested and stained for envelope protein expression using mAb 573 (100) and for Glycogag protein expression using mAb 34 (25, 101). Goat anti-mouse IgM APC and Goat anti-mouse IgG (H+L) APC were used to detect bound mAb 573 and mAb 34, respectively. DFJ8 cells transduced with FMLV-ΔEnv, and FMLV-ΔGlycogag were used as controls for staining. After confirming knockout of Env and Glycogag, transduced DFJ8 cells were cultured for 7 days to amplify VLP, which were subsequently purified by ultracentrifugation as described above.

### ERV and VLP quantification

10 μL of concentrated ERV or VLP were lysed in 990 μL of TRIzol reagent (Invitrogen) and homogenized by vigorous vortexing. 200 μL of chloroform were added to each sample, followed by vortexing. The aqueous layer was isolated by centrifugation at 12,000 × g for 15 min at 4°C, and 500 μL of isopropanol and 5 μg of glycogen were added to the aqueous phase. RNA was then pelleted by centrifugation at 12,000 × g for 10 min at 4°C and washed twice with ice-cold 75% ethanol. After removing all liquid, the RNA pellets were dried in a tissue culture hood and resuspended in 10 μL of RNase-free water. Viral cDNAs were synthesized using the SuperScript III Cells Directed cDNA synthesis Kit (Invitrogen) with all isolated RNA, and qPCR was performed as described above using 1 μL of the cDNA reaction (undiluted, 1/10 diluted and 1/100 diluted) and the MLV_Pol primer set (sequence listed in Table S3). A standard Curve for viral RNA copy numbers was generated by qPCR of the pUC19-ERV plasmid encoding full-length ERV sequence (7), and viral RNA copy number and concentration were quantified according to this standard curve.

### Virions biotinylation and streptavidin conjugation

The following protocol was developed for the preparation of 400 μL of 10^8^ tagged ERV or VLP (4.14 × 10^−10^ mM), which were freshly prepared prior to each ERV-baiting assay. The calculated volume of virions was diluted to a final volume of 1 mL with sterile PBS. The diluted virions were added to a 15 mL 10,000 MWCO centrifugal filter unit (Millipore) containing 11 mL of PBS and centrifuged at 5000 × g for 50 min at RT to remove OptiMEM from the solution. The residual solution in the unit containing concentrated virions was transferred to a new Eppendorf tube. The filter unit was washed with 200 μL of PBS, which was added to the same Eppendorf tube. EZ-link^™^ Sulfo-NHS Biotin (ThermoFisher) was reconstituted with RNase-free water and 16.5 μL of 5 mM biotin was added to PBS containing virions to achieve 5 × 10^5^ molar excess of biotin for each mole of virions. Additional PBS was added to bring the total volume to 400 μL and the reaction was incubated at RT for 1 hour. After biotinylation, the 400 μL reaction was transferred to a new 10,000 MWCO centrifugal filter unit containing 11.6 mL of 100 mM glycine in PBS and incubated for 30 min at RT to quench unconjugated biotin. Samples were centrifuged at 5000 × g for 30 min and washed twice with 12 mL of PBS to remove excess unconjugated biotin and glycine. The residual solution containing biotinylated virions was transferred to a new Eppendorf tube, and the filter unit was washed with PBS as above to capture any remaining virions in the filter. The final volume of biotinylated virions was adjusted to 365 μL using PBS, and 35 μL of 1 mg/mL PE-SA (BioLegend) or PE-SA-AF647 (Invitrogen) were added to the ERV- and VLP-containing solution, respectively. The reaction was incubated covered at RT for 1 hour. The tagged virions in solution were then transferred to a new 2 mL Eppendorf tube and 10% FBS in PBS was added to bring the total volume to 1.5 mL. The sample was underlaid with 200 μL of 15% sucrose in PBS and centrifuged at 12,000 × rpm at 4°C for 2 hours to remove excess SA-fluorophore. The pelleted virions were resuspended in 400 μL of 10% FBS in PBS.

### ERV-baiting assay

For each sample, 500,000 splenocytes or peritoneal cells were plated in 100 μL of 10% FBS in PBS in a round bottom 96-well-plate. Cells were incubated with 25 μL of SA-PE-AF647 tagged VLP for 30 min at RT while covered by foil, and then equilibrated to 4 °C before addition of ERV. Next, 25 μL of ice-cold SA-PE tagged ERV were added to each sample and the total volume was adjusted to 200 μL with 10% FBS in PBS. The plate was then incubated covered at 4°C for 30 min. After incubation, all samples were washed three times with 200 μL of ice-cold PBS before staining with anti-mouse CD3-BV605, CD19-BV421, CD5-FITC, CD23-BV510, CD21/35-APC/Cy7 (BioLegend, listed in Table S3) for 15 min at 4°C while covered by foil. Two washes with 200 μL PBS were performed between all steps. Flow cytometry data were analyzed with FlowJo. For further analysis, targeted population of cells were sorted into RPMI media by performing FACS on the FACSAria cell sorter at Yale Flow Cytometry Facility. Sorted cells were immediately processed for 10X sequencing or for *in vitro* culture as described in this study.

### Recombinant monoclonal antibody production

From the ERV-reactive B-1 BCR repertoire, V(D)J sequences encoded by IGHV1–53, IGHV3–6, IGHV6–3, and IGHV7–3 were extracted. A consensus sequence of the largest clones from each IGHV gene was generated for recombinant DNA synthesis (IDT). Monoclonal antibodies were produced according to a previously published protocol (102). Heavy chain V(D)J sequences were cloned into pRVL-2 vectors (γ2c) (Addgene) and light chain sequences were cloned into pRVL-1 vectors (κ) (Addgene). Heavy and light chain pairings were as follows: IGHV1–53 heavy chain with IGKV4 light chain, IGHV3–6 heavy chain with IGLV1 light chain, IGHV6–3 heavy chain with IGKV14 light chain, and IGH7–3 heavy chain with IGLV1 light chain. Heavy and light chain expression plasmids (100 μg total) were transfected at a ratio of 2:1 into 100 mL of Expi293F^™^ cell culture (Gibco) using the ExpiFectamine^™^ 293 Transfection Kit (ThermoFisher) according to the manufacturer’s manual. On day 7 after transfection, supernatants were harvested and adjusted to the salt concentration of 100 nM NaCl and 20 mM Tris-HCl (pH8), followed by 2-hour RT-incubation with 2 mL of Protein G agarose (ThermoFisher) with gentle rotation. The mixtures were then loaded onto gravity-flow Econo-Pac^®^ Chromatography Columns (Bio-Rad). Packed beads were washed twice with 5X CV of PBS. Bound antibodies were eluted with 4 mL of elution buffer (100 mM glycine, 100 mM NaCl, pH 2.7) into 15 mL collection tubes each containing 1 mL of neutralization buffer (1 M Tris-HCl, pH 7.6). Elutes were concentrated by centrifugation using 30,000 MWCO filter units (GE Healthcare). Buffer exchange was performed in the same filter unit by adding three times the elution volume of PBS. The concentration of mAb was determined by absorbance at 280nm. To produce domain-swapped mAb, CDR3 sequences were exchanged by seamless DNA cloning using Gibson Assembly^®^ Master Mix (NEB). mAb production was scaled down by using 2.5 mL Expi293F^™^ cell culture. Purification of antibodies was performed in Poly-Prep^®^ Chromatography Columns (Bio-Rad).

### Quantification and statistical analysis

For each plot, values were plotted as individual data points, if applicable, with mean and standard deviation (S.D.) calculated for the respective measured parameter. Statistical analyses were conducted by GraphPad Prism software using one-way or two-way ANOVA with Šídák’s multiple comparisons tests, either within each group or using the mean value of the negative control. Paired and unpaired t-tests with Holm- Šídák’s multiple comparisons were performed when appropriate. All statistical analyses are indicated in the respective figure legend. Quantification methods for RT-qPCR, ELISA, and flow cytometry are described in the methods. In the experiments monitoring for infectious ERV emergence (Figure 1B, Figure S1, and Figure S7C), sample sizes were determined by the number of offspring from each breeding. Individual data points are represented and pooled from more than three independent experiments performed with several generations of mice. In all pooled experiments WT and TLR7^−/−^ mice were included as the negative and positive controls. In Figure 3 and Figure 4, libraries were generated and sequenced once. The sequencing matrix was summarized in Table S1 and Table S2. For Figure S8B and Figure S8C, data points represent one independent experiment. For other figures, plots are representative of two or three independent experiments. Except for cleaning of sequencing results as described in these methods, no other exclusion criteria were applied to any results.

## Supplementary Material

add6608_SupplementalMaterial_v4

Raw data file

## Data Availability

All data needed to evaluate the conclusions in the paper are present in the paper or the Supplementary Materials. Tabulated underlying data for all figures can be found in supplementary data file S1. ERV-reactive B-1 and total naïve B-1 single-cell immune profiling data are available through the accession number GSE207247. The identifiers for all commercial antibodies and reagents were summarized in Table S3. Further information and requests for resources and reagents should be directed to and will be fulfilled by the Corresponding Author, Akiko Iwasaki (akiko.iwasaki@yale.edu).
