# Supplementary material for: A B cell screen against endogenous retroviruses identifies glycan-reactive IgM that recognize a broad array of enveloped viruses
Source: Sci Immunol. Author manuscript; Available in PMC 2025 Apr 2. (PMC11962862; doi:10.1126/sciimmunol.add6608)
Supplement: add6608_SupplementalMaterial_v4 [file NIHMS2066005-supplement-add6608_SupplementalMaterial_v4.docx]

**Supplementary materials and methods**

**Cell isolation and culture**

To isolate PBMC, mice were anesthetized with isoflurane, and blood was collected by retro-orbital bleed using heparinized tubes (Fisher Scientific). Collected blood was directly resuspended in 8 mM EDTA in PBS. Red blood cells were lysed with ACK lysis buffer (150mM NH_4_Cl, 1 M KHCO_3_, 0.1mM EDTA, pH 7.4) and nucleated cells were pelleted by centrifugation and then washed twice with PBS. To isolate RNA, 350 µL of RLT buffer (QIAGEN) containing b-mercaptoethanol were added for cells isolated from 100 µL of whole blood.

To isolate splenocytes, the spleens were isolated from euthanized mice and dissociated through 70 µm filters in 5% FBS in RPMI media. Red blood cells were lysed by ACK lysis buffer and cells were pelleted by centrifugation. Cell debris and connective tissue were removed using a 40 µM filter, and splenocytes were washed with 5% FBS in PBS. Isolation of CD4^+^ T cells or bulk B cells was performed using EasySep™ Mouse CD4 T cell isolation kit (STEMCELL) or EasySep™ Mouse Pan-B Cell Isolation Kit (STEMCELL) according to the manufacturer’s manuals. Cells were resuspended in 10% FBS in PBS for subsequent use in the ERV-baiting assay, or in RPMI media for subsequent *in vitro* culture.

To isolate peritoneal cells, the peritoneal wash was obtained by injecting 3-6 mL of 2% FBS with 2 mM EDTA in PBS into the peritoneal cavity. Following gentle shaking, the whole fluid was withdrawn by syringe and placed into 15 mL conical tubes. Peritoneal cells were then pelleted by centrifugation and washed with PBS. Isolation of bulk B cells was performed using the EasySep™ Mouse Pan-B Cell Isolation Kit (STEMCELL) according to the manufacturer’s manuals. Cells were resuspended in 10% FBS in PBS for use in the ERV-baiting assay or in RPMI media for *in vitro* culture.

To culture isolated B cells, 1,000,000 splenic B cells or peritoneal B cells were plated in 1mL RPMI media in a 12-well-plate. For FACS-sorted B cells, 10,000 cells were plated in 200 µL RPMI media in a 96-well-plate. Where applicable, ligands for innate sensors were added to a final concentration as followed: Pam3CSK 0.5 µg/mL; LPS 5 µg/mL; R848 1 µg/mL; CpG 2.5 µg/mL; Poly(I:C) 10 µg/mL; SLR14 10 µg/mL (for SLR14, 2 µL/well of lipofectamine were added in addition to the ligands) (sources are listed in Table S3). After five days of incubation, supernatants were harvested and anti-Emv2 Env antibodies were measured by ELISA, as described in this study.

**Reverse transcription-quantitative polymerase chain reaction (RT-qPCR)**

RNA was isolated from peripheral blood using the RNeasy mini kit (QIAGEN) according to the manufacturer’s manual with the DNA digestion step included. Reverse transcription of isolated RNA was performed using the iScript™ cDNA Synthesis Kit (Bio-rad) in 40 µL reactions. Quantitative PCR was performed using iTaq™ Universal SYBR® Green Supermix (Bio-rad) in 10 µL reactions in triplicate. Each PCR reaction contained 30ng cDNA and primers were used at a final concentration of 0.225 µM. Primer sets used in this study are listed in Table S3.

**Enzyme-linked immunosorbent assays**

96-well EIA/RIA plates (Corning) were coated at 4˚C overnight with 1 µg/mL purified recombinant proteins or ${10}^{6}$ pfu/mL virions in carbonate buffer at a final volume of 100 µL per well in duplicate. Wells coated with carbonate buffer only (empty wells) were used to measure background signals. Plates were blocked with 5% fetal bovine serum (FBS) in phosphate buffer saline (PBS) for 1 hour at room temperature (RT). In all ELISA conducted with recombinant mAb and HGAC mAb, plates were blocked with the Carbo-Free Blocking solution (Vector Lab) to reduce background binding to FBS-derived glycans. Primary incubations were conducted at 4˚C overnight with diluted sera (1/50 dilution), cell culture supernatant (undiluted), or mAb (3 µg/mL) in 100 µL blocking solution per well. Following primary adsorption, bound antibodies were detected by alkaline-phosphatase (HRP)-conjugated mouse Ig isotype-specific secondary antibodies (Southern Biotechnology) at a final concentration of 1 µg/mL in 100 µL blocking solution at RT for 1 hour. Following TMB substrate development the plate were measured by 450nm absorbance (with 490nm absorbance subtracted). Background signals of empty wells following primary and secondary incubation were further subtracted to normalize for background binding in each sample. For detection of the total antibody input signal, the plates were coated with 0.1 µg of unlabeled anti-mouse Ig antibody (Southern Biotechnology) in 100 µL carbonate buffer followed by primary and secondary incubation. In the competitive ELISA, mAb solution (3 µg/mL) was preabsorbed at the indicated concentration of monosaccharide (0 mM – 200 mM) at RT for 2 hours, and then was applied to the antigen-coated plate, followed by secondary antibody incubation.

For measurement of GlcNAc by lectins, plates were coated with purified recombinant protein at a final concentration of 3 µg/mL at 4˚C overnight. Plates were blocked with the Carbo-Free blocking solution. Biotinylated lectins (Vector Lab) at a final concentration of 5 µg/mL in blocking buffer were applied to wells and the plates were incubated at RT for 30min. For each lectin, the negative control was made by pre-adsorption of lectins with 200 mM of free GlcNAc at RT for 2 hours. Lectins without blocking sugars were also incubated at RT for 2 hours to normalize for lectin activity. Bound lectins were detected using VECTASTAIN® Elite ABC-HRP Kit (Vector Lab) according to the manufacturer’s manuals. For each lectin, the absorbance of respective negative controls was subtracted.

**Recombinant protein production**

Env Glycogag sequences were cloned from *Emv2* viral sequence (*7*) using Q5^®^ High-Fidelity 2X Master Mix (NEB) with overlapping arms for inserting into linearized pEZT expression vectors (kindly gifted by Aaron Ring, Yale University) using Gibson Assembly® Master Mix (NEB). Env SU(M) encodes for a protein starting from the Methionine (1) of Env, generating proteins containing the predicted transmembrane N-terminal domain. Env SU(V) encodes for a protein starting from the Valine (50) of Env, generating proteins lacking the N-terminal transmembrane helix. Predicted transmembrane helices at the C-terminal were not included in either Env SU construct. 100 mL of Expi293F™ (Gibco) culture were transduced with 100 µg of Env SU or Glycogag expression vectors using ExpiFectamine™ 293 Transfection Kit (ThermoFisher) according to the manufacturer’s manuals. Cell culture supernatants were harvested on day 4 post-transduction and were adjusted to the salt concentration of 300 nM NaCl and 20 mM Tris-HCl (pH 8) at a final volume of twice the original volume. 1 mL of Ni-NTA Agarose beads (QIAGEN) were incubated with supernatants while stirring at 4˚C for 3 hours. The mixtures were loaded onto gravity-flow Econo-Pac^®^ Chromatography Columns (Bio-Rad). Packed beads were washed with 5X column volumes (CV) of PBS and 5X CV of 20 mM imidazole in PBS. Bound proteins were eluted with 250 mM imidazole in PBS. The elutes were concentrated by centrifugation using 30,000 MWCO filter units (GE Healthcare). Buffer exchange was performed in the same filter unit by adding 3X elution volume of PBS. The concentration of purified proteins was determined from the absorbance at 280nm.

**10X Single-cell sequencing and analysis**

Sorted ERV-reactive B-1 cells and total naïve B-1 cells were counted and loaded into the Chromium Controller (10X Genomics) for single-cell partitioning and barcoding. Single-cell V(D)J libraries were generated using the Chromium Single Cell V(D)J Reagent kit (10X Genomics) while single-cell gene expression libraries were generated using the Chromium Single Cell 5’ Reagent kit (10X Genomics), per the manufacturer’s instruction. Libraries were sequenced on the NovaSeq 6000 Sequencing System (Illumina). Single-cell V(D)J libraries were sequenced with 2 $\times$ 150 bp paired-end reads and single-cell gene expression libraries were sequenced with 2 $\times$ 100 bp paired-end reads. Details for the sequencing matrix are listed in the supplemental information (Table S1 and S2). FASTQ sequences were generated, demultiplexed and aligned to the reference genome using the Cell Ranger package (10X Genomics). Gene expression was further analyzed using Seurat V3 (*103*). Cells that have unique feature counts below 200 or over 2,500, or that contain more than 5% mitochondrial counts, were removed. Gene expression values were log-normalized using the function *NormalizedData*. Genes mapped to immunoglobulin loci were removed and data were scaled before identifying highly variable genes using *FindVariableGenes*. Linear dimensional reduction was performed based on scaled data and clusters were identified using the functions *FindNeighbors* and *Findclusters* based on top 10 principal components with resolution set to 0.5. Clusters exhibiting T cell features were removed before final linear dimensional reduction and clusters calling. Clusters visualizations were performed with UMAP using the top 10 principal components. Gene ontology analysis was performed using the package Enrichr (*104*), based on GO_Biological_Process_2021.

**B cell receptor repertoire analysis**

Reconstructed V(D)J sequences from Cell Ranger output were further analyzed by using the Change-O package (*105*). V(D)J germline assignments were performed with IgBLAST (*106*) using the IMGT reference gene database (*107*). Cells with multiple V(D)J sequences were assigned to the most abundant V(D)J sequence by UMI count. Cells with non-functional V(D)J were filtered prior to clonal grouping. Sequences were first grouped by IGHV and IGHJ gene annotations and junction length, and nucleotide hamming distance was calculated. Grouping of B cell clones was additionally performed by setting the hamming distance threshold to 0.16. Clonal groups were corrected based on light chain data. Multiple CDR3 properties and SHM rates were calculated using the packages Alakazam and SHazaM (*105*). IGHV and IGHJ gene selection were visualized by treemap, in which each output square was first grouped by IGHV genes and then further subdivided according to IGHJ selection. The size of each square represents the UMI counts. V(D)J assignment and clonal grouping were integrated with gene expression data according to cell barcodes. Clonal types characterization based on frequency ranges and clonal overlap calculation between clusters were performed using the ScRepertoire package (*108*).

**Western Blotting**

Purified recombinant proteins were denatured and deglycosylated by β-N-Acetylglucosaminidase S (NEB), PNGase F (NEB) or O-Glycosidase & Neuraminidase Bundle (NEB) according to the manufacturer’s protocols. Samples were then subjected to electrophoresis in 12% polyacrylamide gel followed by transfer onto PVDF membranes. PVDF blots were probed with HRP conjugated anti-His tag antibody (Cell signaling) (1/1000 dilution), or with 3 µg/mL monoclonal antibody followed by detection using a 1/1000 dilution of HRP-conjugated anti-mouse IgG secondary antibody (Southern Biotech). Blocking of the membrane and antibody dilutions were all performed using the Carbo-free blocking solution (Vector Lab). Blots were developed using Pierce™ ECL Western Blotting Substrate or SuperSignal™ West Pico PLUS Chemiluminescent Substrate (ThermoFisher).

**Mice in vivo stimulation**

Mice aged 6-8 weeks were i.p. injected with R848 (InvivoGen), LPS (InvivoGen) or CpG (TriLink BioTechnologies) at a dose of 18 µg in 100 µL of PBS per mouse. Sera were collected by retro-orbital bleed prior to stimulation and then daily for six days. Mice aged 10 weeks were i.p. injected with ${10}^{6}$ ERV particles or ${5\times10}^{5}$ isolated *Tlr7^-/-^* T cells (as described in cell isolation method) in 100 µL of PBS per mouse or i.n. infected with 30 pfu of PR8 (kindly gifted by Hideki Hasegawa, National Institute of Infectious Diseases, Tokyo) in 100 µL PBS per mouse. Sera were collected by retro-orbital bleed on day 0 and on day 6 following stimulation.

**ERV in vitro infection**

DFJ8 cells were seeded at ${1\times10}^{5}$ cells per well with 500 µL DMEM in each well of a 24-well-plate. On the day of infection, ${5\times10}^{5}$ of ERV particles (M.O.I. = 5) were incubated with 2.5 µg of mAb, with or without 2.5 µL of *Rag1^-/-^ MD4 Tg* serum in OptiMEM in a total volume of 50 µL. After a 1-hour incubation at 37˚C, DMEM in the 24-well-plate were removed and replaced by 50 µL of OptiMEM, each 50 µL mixture containing ERV and mAb was then added to a DFJ8 cells-containing well to reach a final volume of 100 µL. After a 1-hour incubation at 37˚C, the supernatant was removed and 500 µL of fresh pre-warmed DMEM were added. At 48 hours post-infection, cells were harvested for staining with mAb 573 to quantify the percentage of infected cells.

**Mouse in vivo infection**

To infect mice with herpes simplex virus-2 (HSV-2), mice were injected subcutaneously in the neck scruff with 2mg per mouse of Depo-Provera (GE Healthcare) five days prior HSV-2 infection. On the day of infection, mice were swabbed with PBS-soaked calcium alginate in vaginal track and then infected intravaginally with 10000 pfu of WT HSV-2 (186syn+ strain) in 10ul. Vaginal fluids were collected on day 1 and 2 post infection. The vaginal cavity was swabbed by PBS-soaked calcium alginate and washed with 50ul of sterile PBS. Both of swab and washes were collected in 950ul ABC buffer (0.5mM MgCl_2_6H_2_0,0.9mM CaCl_2_2H_2_O, 1% glucose, 5% HI FBS in PBS). Viral titers of vaginal washes were obtained by plaque assay as previously described (*109*).

To infect mice with SARS-CoV-2, 12-week-old K18-hACE2 mice were used. SARS-CoV-2 infection was performed as previously described (*110, 111*).Briefly, mice were first anesthetized using 30% v/v isoflurane diluted in propylene glycol. Following anaesthetization, mice were intranasally infected with 1×10^4^ PFU SARS-CoV-2 in 30 μl inoculation volume using a pipette. Two days post infection, mice were euthanized with 100% isoflurane for bronchoalveolar lavage fluid (BALF) collection. After exposing the trachea, the lungs were slowly inflated with 1 ml PBS through intratracheal instillation. Inflation was repeated three times.

**
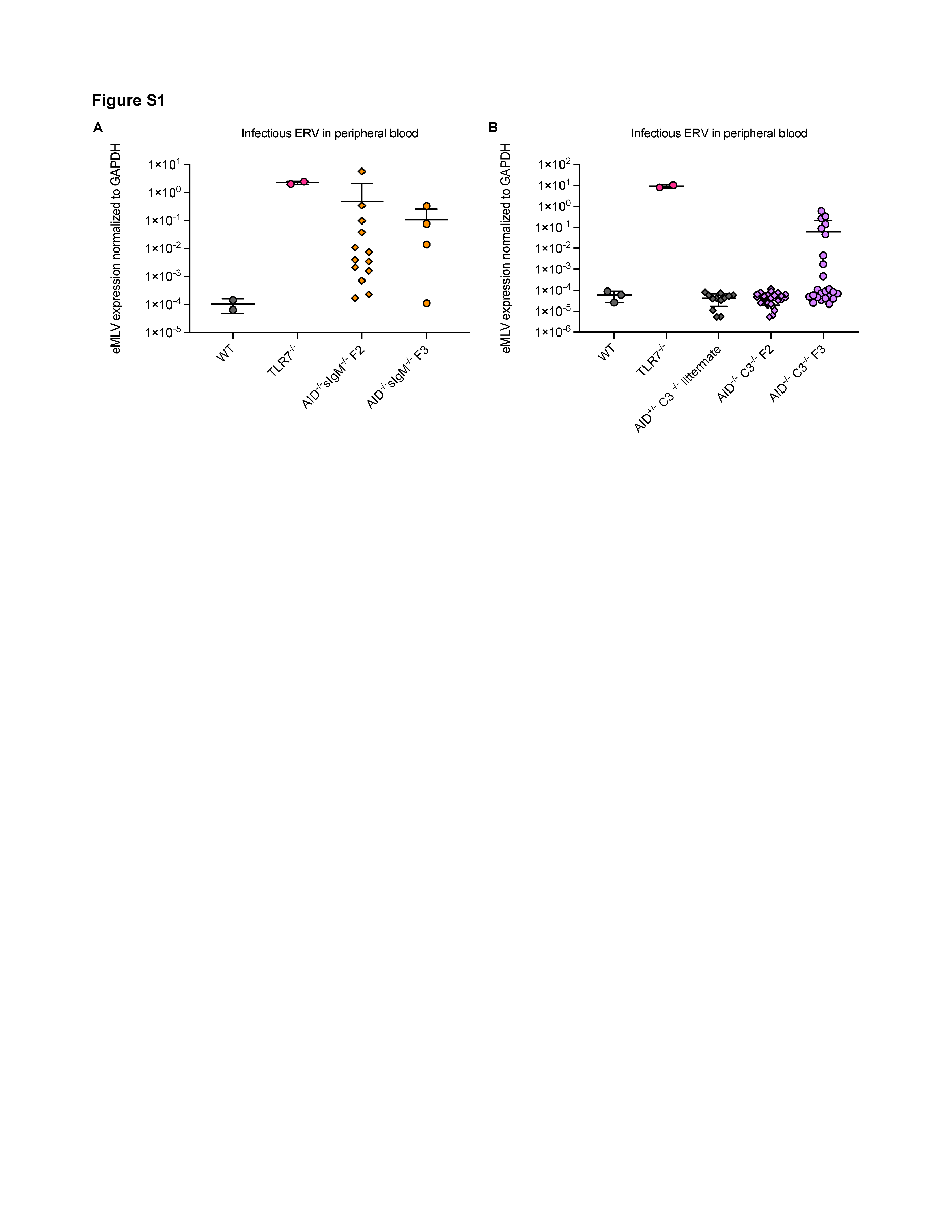
**

**Figure S1 (Related to Figure 1). Emergence of ERV were established in mice with deficiency in antibody secretion and in effector molecules.**

(A) and (B) RT-qPCR of RNA isolated from the PBMC of WT mice or from mice with indicated genotypes measuring spliced ecotropic Env (Emv2) transcription. Values are normalized to internal GAPDH expression. *Tlr7^-/-^* mice were bred as homozygotes for over 10 generations. Generation bred as homozygotes of *AID^-/-^sIgM^-/-^* and *AID^-/-^C3^-/-^* mice were indicated on the x-axis. Each data point represents an individual mouse.

**
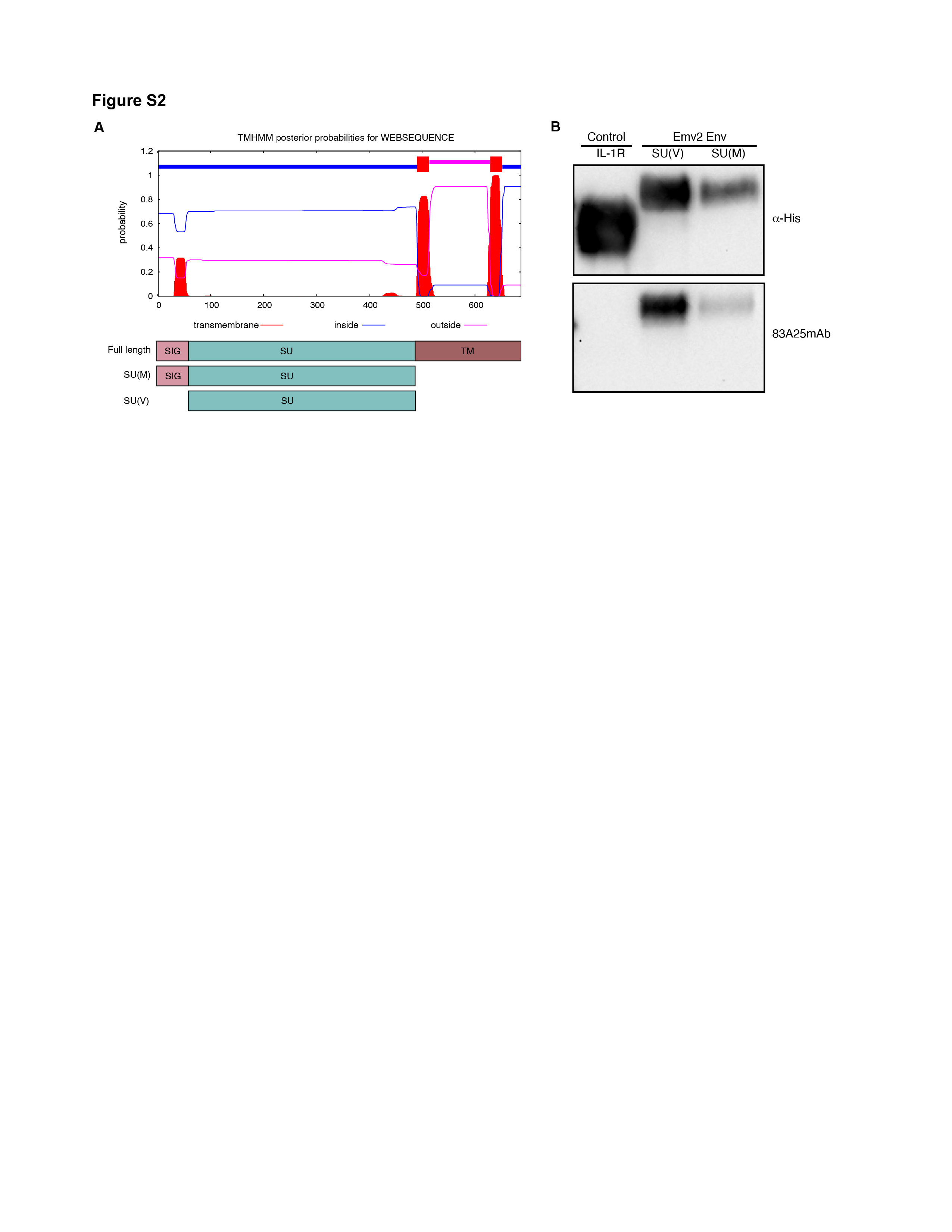
**

**Figure S2 (Related to Figure 1). Generation of recombinant Emv2 Env surface unit retaining epitopes for anti-Env antibody recognition.**

1. Predicted transmembrane a-helices in the full-length Emv2 Env sequence (upper panel) and schematics of the full-length Env containing predicted surface unit (SU), transmembrane domain (TM) and signal peptide (SIG), the SU(M) containing SIG and SU, and the SU(V) containing SU.
2. Western blot of purified recombinant SU(V) and SU(M) proteins detected using HRP-conjugated anti-His Ab or using mAb 83A25 & HRP-conjugated anti-Rat IgG secondary Ab. Recombinant IL-1R proteins produced in the same expression system are the positive control for anti-His detection and the negative control for mAb 83A25 detection.

**
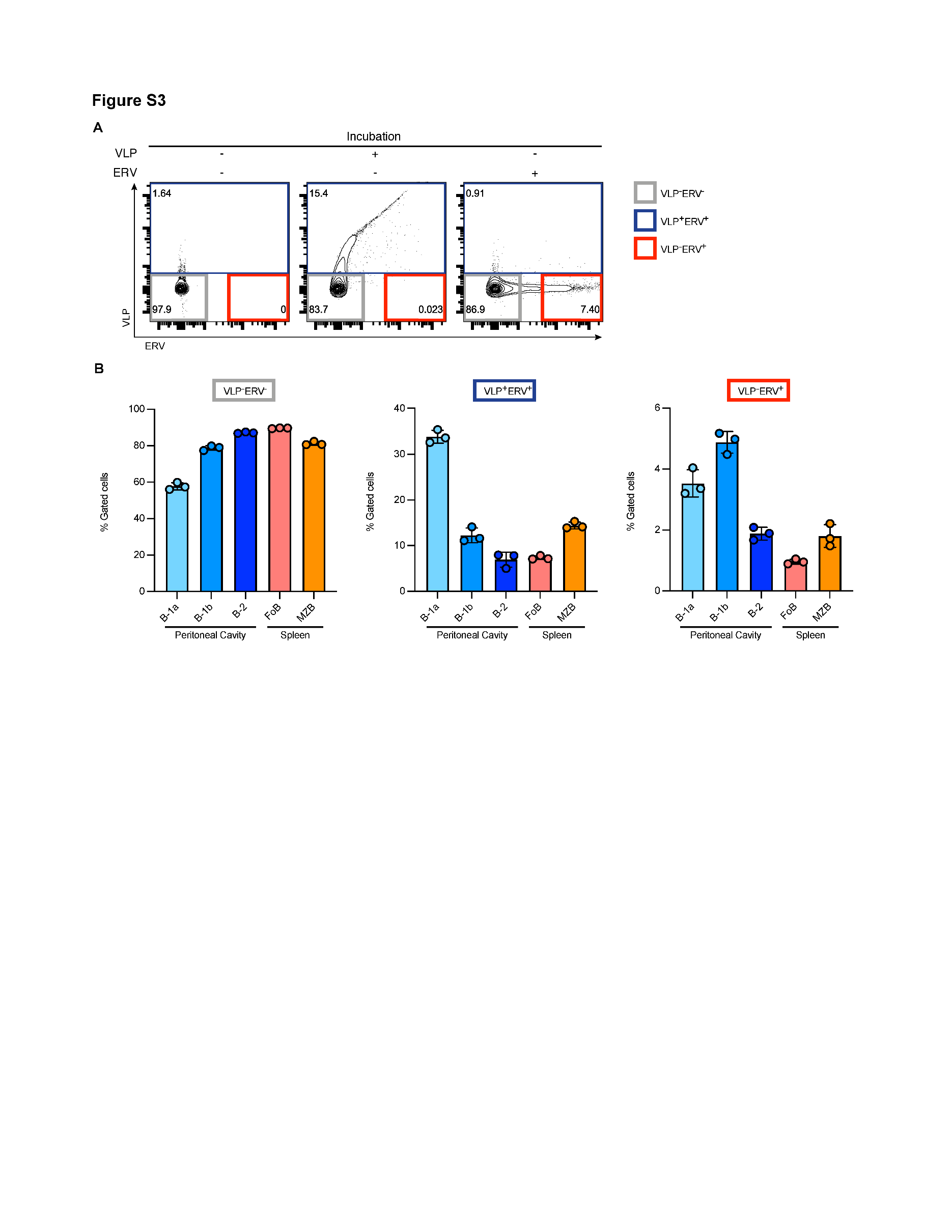
**

**Figure S3 (Related to Figure 2). Fluorescence-tagged ERV-baiting strategy enables detection of ERV-reactive B cells in pre-immune splenic and peritoneal compartments.**

1. Representative flow cytometry plots of peritoneal B cells without virions incubation, or peritoneal B cells incubated with tagged VLP, or tagged ERV, as indicated at the top of the plot. Gated populations include: AF647^-^PE^-^ or VLP^-^ERV^-^ (grey); AF647^+^PE^+^ or VLP^+^ERV^+^ (blue); AF647^-^PE^Hi^ or VLP^-^ERV^+^ (red). The percentage of gated cells is listed in each gate.
2. The percentage of VLP^-^ERV^-^ cells (left), VLP^+^ERV^+^ cells (middle) and VLP^-^ERV^+^ cells (right) plotted for comparison between peritoneal B-1a, peritoneal B-1b, peritoneal B-2 cells, splenic FoB and splenic MZB cells. Each data point represents an individual mouse.

**
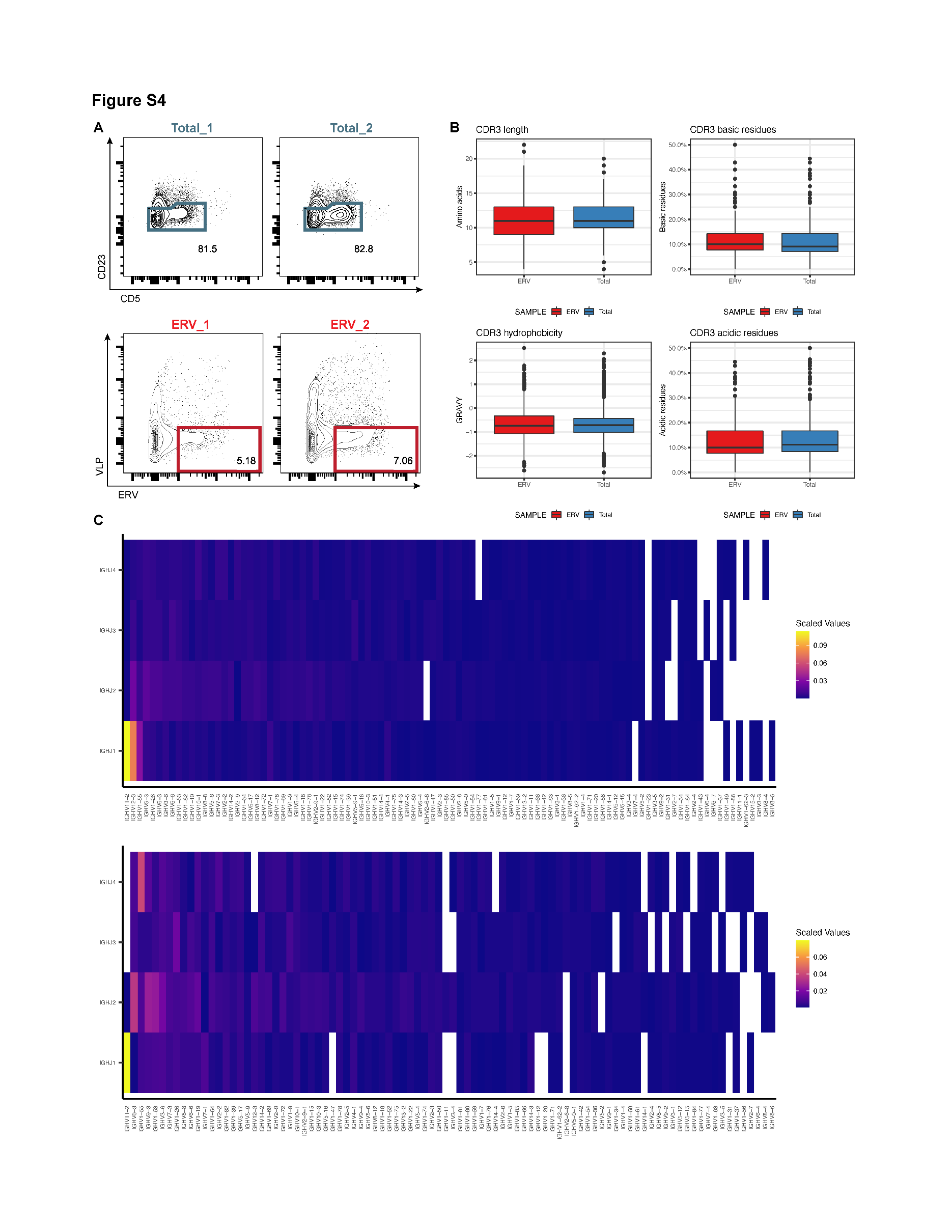
**

**Figure S4 (Related to Figure 3). CDR3 length distribution but no other CDR3 property was different in ERV-reactive B-1 repertoire compared to total B-1 repertoire.**

1. Gating schema for the FACS-sorted total naïve peritoneal repertoire (top, blue gate) and the ERV-reactive repertoire (bottom, red gate). The percentage of gated cells is listed near or in gate for each plot. each plot represents the cells from an individual mouse.
2. Calculated CDR3 length, CDR3 hydrophobicity, percentage of basic residues across CDR3, percentage of acidic residues across CDR3, of the total and the ERV-reactive peritoneal B-1 repertoires. Sequences are pooled from two individual mice from each group.
3. Heatmap representation of the relative frequencies of each V and J recombination in the total B-1 repertoire (top) and the ERV-reactive B-1 repertoire (bottom). IGHV gene names are listed on the x-axis and IGHJ gene names are listed on the y-axis. The color scale is shown in each panel. White boxes indicate the absence of the corresponded recombination.

**
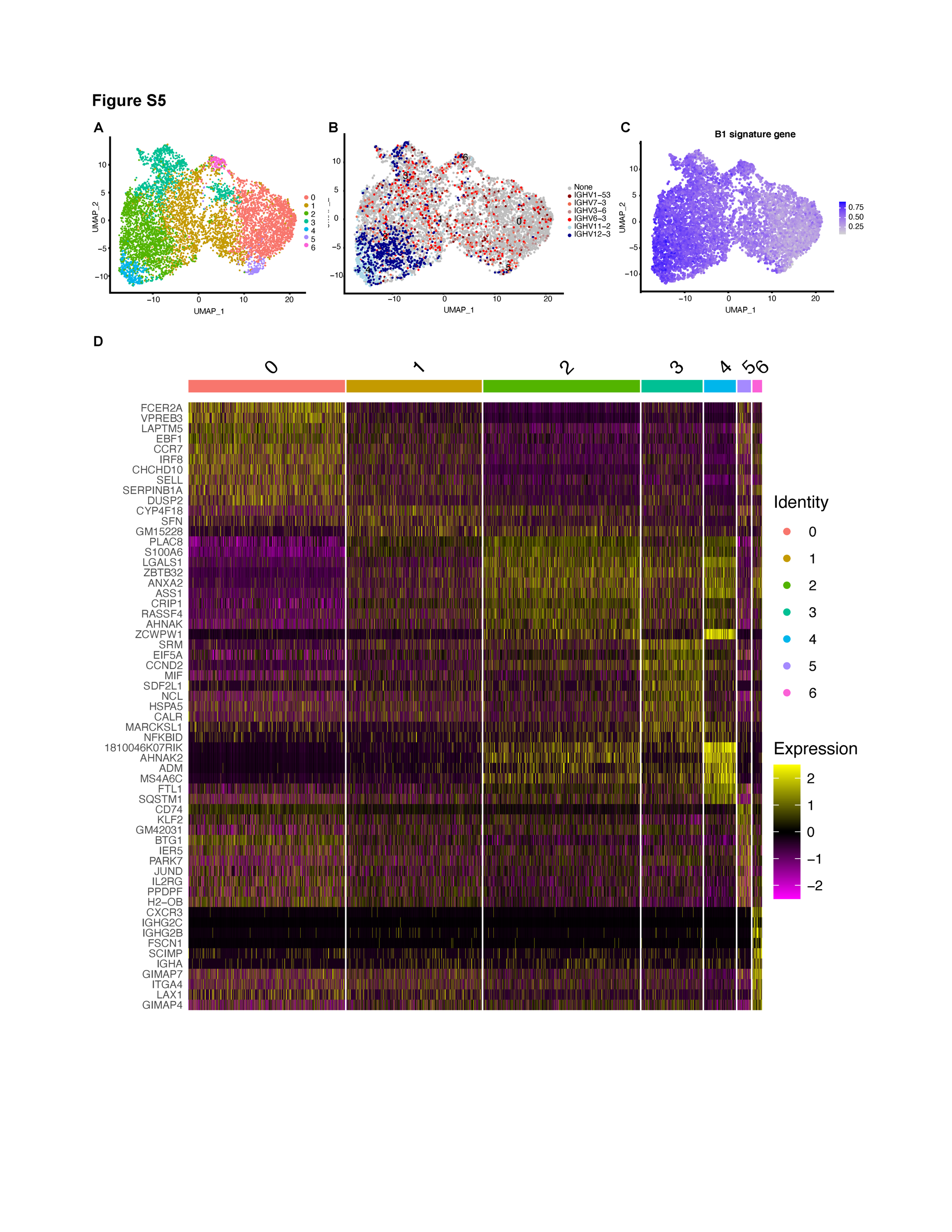
**

**Figure S5 (Related to Figure 4). Cluster 3 with expanded clones were enriched in ERV-reactive peritoneal B-1 repertoire and expressed genes related to cell survival.**

1. UMAP representation of merged datasets of B-1 cells from the total repertoire and B-1 cells from ERV-reactive repertoire with assigned cluster identities listed in the legend.
2. UMAP representation of merged datasets of total and ERV-reactive B-1 cells highlighting cells expressing the listed IGHV genes.
3. UMAP representation of merged datasets of total and ERV-reactive B-1 cells depicting the combined expression level of a list of B-1 cell signature genes. The average expressions of B-1 cell features are subtracted by the aggregated expression of randomly sampled control feature sets.
4. Heatmap displaying the expression of top 10 differentially expressed genes (DEG) in each cluster in merged datasets of total and ERV-reactive B-1 cells. Top 10 DEG (only positive markers) were identified in each cluster compared to all remaining cells. Cluster identities are listed at the top. The color scale of the expression level is shown in the legend. The top 10 DEG were identified by the Wilcoxon rank sum test.

**
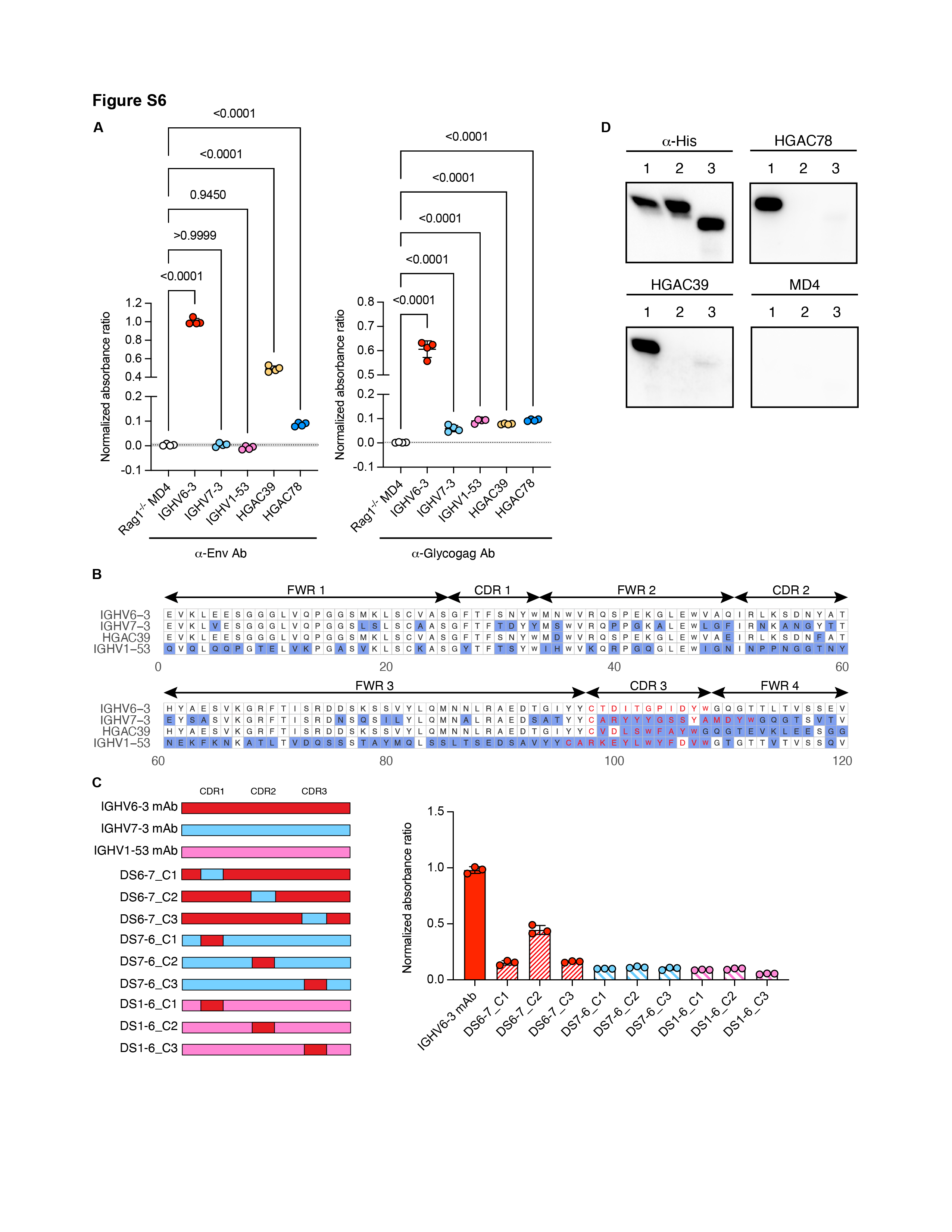
**

**Figure S6 (Related to Figure 5). Monoclonal antibodies encoded by IGHV6-3 recognize Env through terminal GlcNAc.**

1. (Related to Figure 5A) ELISA of purified recombinant monoclonal antibodies measuring the reactivities to ERV Env (left panel) and Glycogag (right panel). Serum from *Rag1^-/-^ MD4 Tg* mice were incubated as the negative control. All values were normalized by ratio to the signal of total antibody input (total antibody was captured by coating the plate with unlabeled anti-mouse Ig Ab, for each antibody, and were detected by the same secondary antibody). Data are plotted as mean ± SD, with individual data points represents technical replicates. Dotted line and grey filled area represent mean ± 2SD of the negative control. P-values were calculated using one-way ANOVA with Šídák’s multiple comparisons test to the mean value of negative control.
2. Amino acid sequence alignment of the listed monoclonal antibodies across variable regions. Boxes colored in blue represent different amino acids compared to IGHV6-3 mAb as the reference sequence. FWRs and CDRs of IGHV6-3 mAb are indicated at the top of the alignment. Amino acid sequence in CDR3 of each mAb is colored in red.
3. Schematic of the domain-swapped antibody sequence is depicted (left). The colors were assigned to original IGHV genes. ELISA of purified recombinant domain-swapped antibodies measuring the reactivities to ERV Env (right). All values were normalized by ratio to the signal of total antibody input (total antibody was captured by coating the plate with unlabeled anti-mouse Ig Ab, for each antibody, and were detected by the same secondary antibody). Data are plotted as mean ± SD, with individual data points represent technical replicates.
4. Western blot analysis of denatured Env (Lane 1, control), b-N-Acetylglucosamine S-treated Env (Lane 2) and PNGase F-treated Env (Lane 3). Bands were detected by using anti-His Ab, HGAC78 (m), HGAC39 (g3) mAb or serum from *Rag1^-/-^ MD4 Tg* mice (negative control).

**
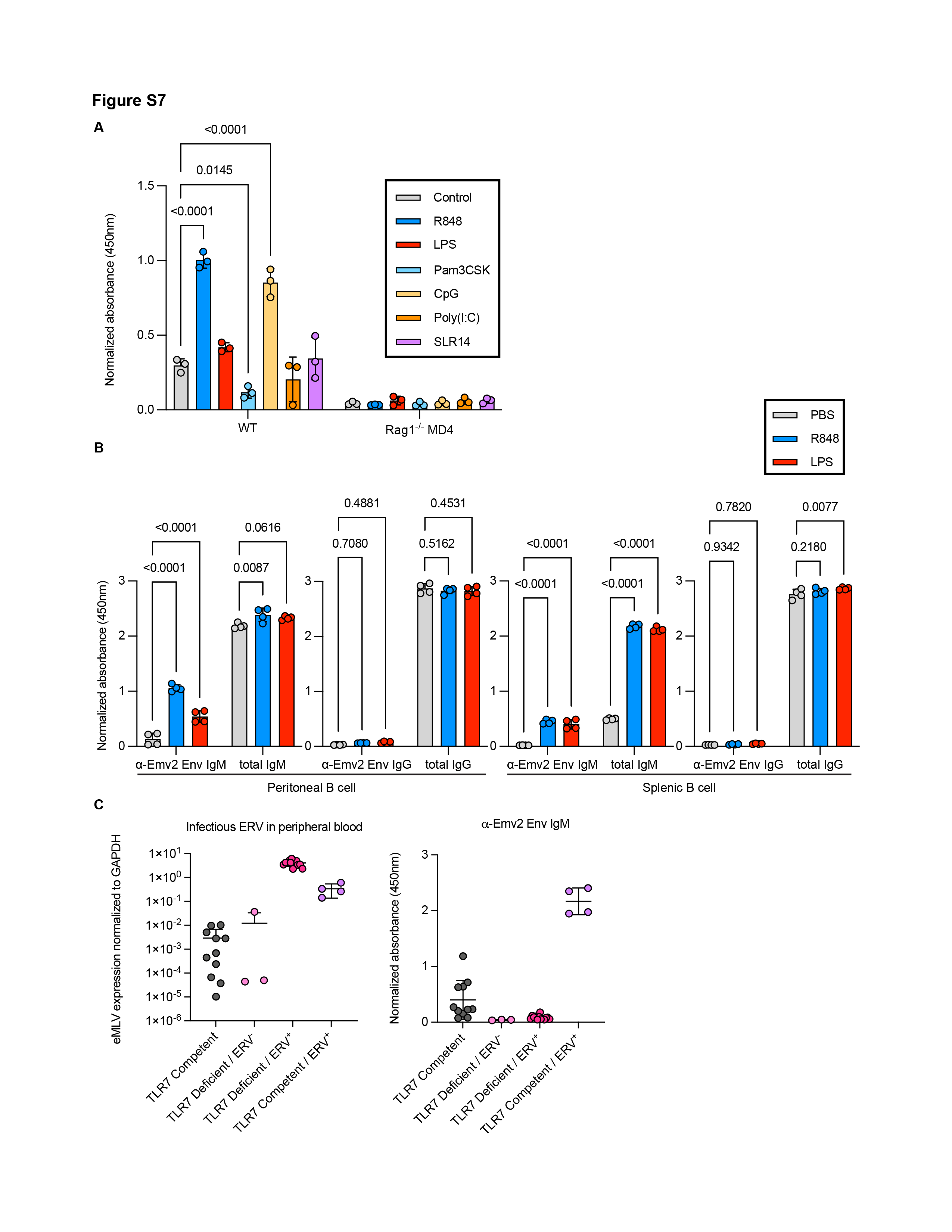
**

**Figure S7 (Related to Figure 6). Secretion of ERV-reactive IgM by B-1 cells was induced by TLR activation.**

1. ELISA of anti-Env IgM in the supernatant of purified peritoneal B cells of WT C57BL/6 mice and *Rag1^-/-^ MD4 Tg* mice (negative control) after five days of cell culture. Synthetic ligands for innate immune sensors were added in the culture as indicated. Data are plotted as mean ± SD, with each data point represents cells pooled from three mice. P-values were calculated using two-way ANOVA with Šídák’s multiple comparisons.
2. ELISA of anti-Env IgM and IgG in the supernatant of purified peritoneal B cells and splenic B cells of WT C57BL/6 mice after five days of cell culture. LPS and R848 were added in the culture as indicated. Data are plotted as mean ± SD, with each data point represents cells pooled from two mice. P-values were calculated using two-way ANOVA with Šídák’s multiple comparisons.
3. RT-qPCR of RNA isolated from the PBMC of TLR7-competent F1 mice (heterozygous littermates), TLR7-deficient F1 mice without ERV viremia, TLR7-deficient F1 mice with ERV viremia, and TLR7-competent mice with ERV viremia (*AID^-/-^C3^-/-^* F3), measuring spliced ecotropic Env transcription (left). Values are normalized to internal GAPDH expression. ELISA of IgM reactive to Env in the serum collected from the same group of mice. Each data point represents an individual mouse.

**
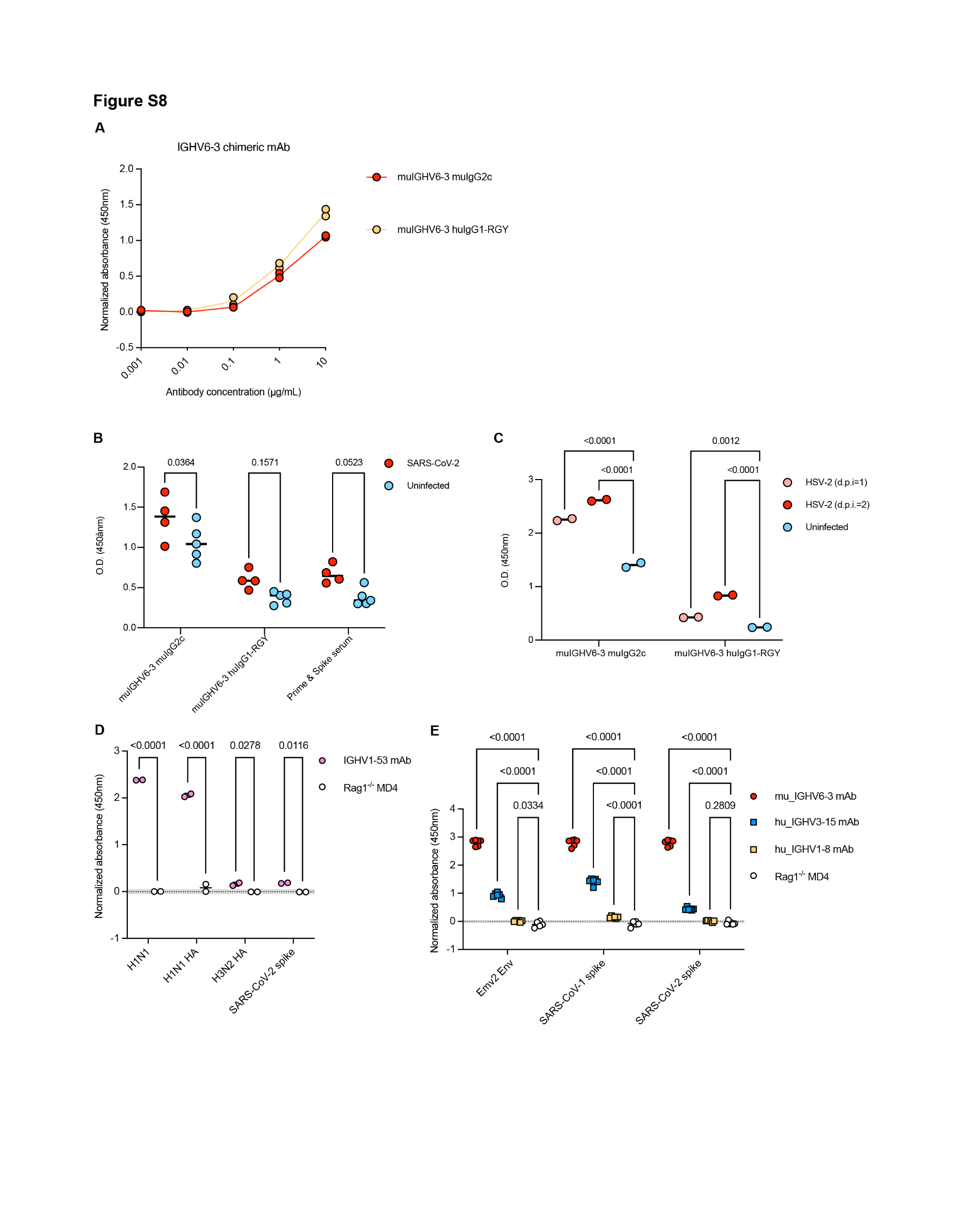
**

**Figure S8 (Related to Figure 7) ERV-reactive germline antibodies recognize different enveloped viruses.**

1. ELISA of purified recombinant murine IGHV6-3-murine IgG2c mAb and murine IGHV6-3-human IgG1-RGY (E345R, E430G, S440Y) chimeric mAb (*112*), measuring the reactivities to Emv2 Env at respective concentrations. Data are plotted as individual data points representing technical replicates.
2. ELISA of purified murine IGHV6-3-murine IgG2c mAb and murine IGHV6-3-human IgG1-RGY mAb measuring the reactivities to the bronchoalveolar lavage fluid (BALF) of SARS-CoV-2 (1×10^4^ pfu) infected mice harvested on Day 2 post-infection. BALF from uninfected mice was used as the negative control. Immune serum from the mice intramuscularly primed and intranasally boosted with SARS-CoV-2 vaccine was used as the positive control. Data are plotted as individual data points representing biological replicates. p-values were calculated using two-way ANOVA with Šídák’s multiple comparisons.
3. ELISA of purified murine IGHV6-3-murine IgG2c mAb and murine IGHV6-3-human IgG1-RGY mAb measuring the reactivities to the vaginal wash of HSV-2 (1×10^4^ pfu) infected mice. Vaginal wash from uninfected mice was used as the negative control. HSV-2-containing vaginal wash were obtained on Day 1 post-infection (coated at 1×10^3^ pfu/well determined by plaque assay) and on Day 2 post-infection (coated at 1×10^5^ pfu/well determined by plaque assay). Data are plotted as individual data points representing biological replicates. p-values were calculated using two-way ANOVA with Šídák’s multiple comparisons.
4. ELISA of murine IGHV1-53 mAb measuring reactivity to viral proteins. Serum from *Rag1^-/-^ MD4 Tg* mice was incubated as the negative control. Data are plotted as mean ± SD, with individual data points representing technical replicates. The dotted line and grey filled area represent the mean ± 2SD of the negative control. P-values were calculated using two-way ANOVA with Šídák’s multiple comparisons.
5. ELISA of human IGHV3-15 mAb measuring reactivity to viral proteins. Serum from *Rag1^-/-^ MD4 Tg* mice was incubated as the negative control. Murine IGHV6-3 mAb was incubated as the positive control. Antibody encoded by IGHV1-8, which is the most abundant IGHV gene in putative human B-1 repertoire is used as an additional control. Data are plotted as mean ± SD, with individual data points representing technical replicates. The dotted line and grey filled area represent the mean ± 2SD of the negative control. P-values were calculated using two-way ANOVA with Šídák’s multiple comparisons.

**Table S1. Matric summary for 5’gene expression single cell profiling.**

| **Sample** | **ERV_1_**  **5GENE** | **ERV_2_**  **5GENE** | **Total_1_**  **5GENE** | **Total_2_**  **5GENE** |
| --- | --- | --- | --- | --- |
| **Estimated Number of Cells** | **2,531** | **1,472** | **5,192** | **5,039** |
| **Mean Reads per Cell** | 21,887 | 32,667 | 29,176 | 29,772 |
| **Median Genes per Cell** | 1,686 | 1,952 | 1,798 | 1,895 |
| **Number of Reads** | 55,396,690 | 48,086,110 | 151,485,136 | 150,023,312 |
| **Valid Barcodes** | 89.20% | 88.30% | 89.00% | 89.30% |
| **Sequencing Saturation** | 56.10% | 64.20% | 65.30% | 65.00% |
| **Q30 Bases in Barcode** | 97.40% | 97.40% | 97.30% | 97.40% |
| **Q30 Bases in RNA Read** | 93.70% | 92.60% | 93.90% | 94.20% |
| **Q30 Bases in Sample Index** | 92.60% | 93.70% | 91.40% | 91.00% |
| **Q30 Bases in UMI** | 97.00% | 97.10% | 97.00% | 97.00% |
| **Reads Mapped to Genome** | 94.00% | 92.20% | 94.30% | 94.60% |
| **Reads Mapped Confidently to Genome** | 88.20% | 86.50% | 88.50% | 88.90% |
| **Reads Mapped Confidently to Intergenic Regions** | 6.60% | 6.70% | 6.90% | 6.70% |
| **Reads Mapped Confidently to Intronic Regions** | 5.40% | 5.30% | 5.00% | 4.90% |
| **Reads Mapped Confidently to Exonic Regions** | 76.10% | 74.50% | 76.70% | 77.30% |
| **Reads Mapped Confidently to Transcriptome** | 69.80% | 68.20% | 70.20% | 70.80% |
| **Reads Mapped Antisense to Gene** | 3.80% | 3.90% | 3.90% | 3.90% |
| **Fraction Reads in Cells** | 98.10% | 98.00% | 98.20% | 98.20% |
| **Total Genes Detected** | 14,301 | 14,041 | 15,565 | 15,765 |
| **Median UMI Counts per Cell** | 5,466 | 6,662 | 5,798 | 6,268 |

**Table S2. Matric summary for V(D)J repertoire single cell profiling.**

|  | **ERV_1_**  **BCR** | **ERV_2_**  **BCR** | **Total_1_**  **BCR** | **Total_2_**  **BCR** |
| --- | --- | --- | --- | --- |
| **Estimated Number of Cells** | **2,370** | **1,437** | **4,837** | **4,825** |
| **Mean Read Pairs per Cell** | 13,996 | 26,761 | 10,770 | 10,803 |
| **Number of Cells With Productive V-J Spanning Pair** | 2,263 | 1,366 | 4,703 | 4,670 |
| **Number of Read Pairs** | 33,171,900 | 38,456,902 | 52,095,333 | 52,129,115 |
| **Valid Barcodes** | 95.70% | 95.90% | 95.80% | 95.90% |
| **Q30 Bases in Barcode** | 97.30% | 97.50% | 97.50% | 97.50% |
| **Q30 Bases in RNA Read 1** | 95.30% | 95.30% | 95.50% | 95.40% |
| **Q30 Bases in Sample Index** | 91.90% | 95.20% | 95.10% | 95.30% |
| **Q30 Bases in UMI** | 97.40% | 97.60% | 97.50% | 97.60% |
| **Reads Mapped to Any V(D)J Gene** | 94.00% | 94.30% | 94.40% | 94.50% |
| **Reads Mapped to IGH** | 16.60% | 14.90% | 17.90% | 17.30% |
| **Reads Mapped to IGK** | 52.90% | 53.50% | 51.40% | 52.80% |
| **Reads Mapped to IGL** | 24.60% | 25.80% | 25.10% | 24.50% |
| **Mean Used Read Pairs per Cell** | 10,254 | 20,714 | 8,061 | 8,353 |
| **Fraction Reads in Cells** | 81.70% | 86.60% | 84.80% | 87.30% |
| **Median IGH UMIs per Cell** | 25 | 23 | 28 | 29 |
| **Median IGK UMIs per Cell** | 71 | 67 | 78 | 79 |
| **Median IGL UMIs per Cell** | 70 | 81 | 95 | 96 |
| **Cells With Productive V-J Spanning Pair** | 95.50% | 95.10% | 97.20% | 96.80% |
| **Cells With Productive V-J Spanning (IGK, IGH) Pair** | 83.20% | 81.10% | 85.10% | 86.20% |
| **Cells With Productive V-J Spanning (IGL, IGH) Pair** | 17.50% | 17.10% | 15.70% | 13.00% |
| **Paired Clonotype Diversity** | 1241.46 | 364.39 | 500.77 | 366.97 |
| **Cells With IGH Contig** | 97.90% | 97.60% | 99.20% | 99.00% |
| **Cells With IGK Contig** | 90.50% | 89.60% | 91.30% | 92.00% |
| **Cells With IGL Contig** | 69.80% | 69.00% | 68.10% | 71.30% |
| **Cells With CDR3-annotated IGH Contig** | 96.50% | 96.20% | 98.20% | 97.70% |
| **Cells With CDR3-annotated IGK Contig** | 87.00% | 85.20% | 87.60% | 89.10% |
| **Cells With CDR3-annotated IGL Contig** | 18.90% | 18.40% | 16.70% | 13.70% |
| **Cells With V-J Spanning IGH Contig** | 97.70% | 97.20% | 99.00% | 98.60% |
| **Cells With V-J Spanning IGK Contig** | 89.70% | 88.60% | 90.90% | 91.40% |
| **Cells With V-J Spanning IGL Contig** | 21.70% | 20.90% | 19.40% | 16.60% |
| **Cells With Productive IGH Contig** | 95.90% | 95.30% | 97.60% | 97.00% |
| **Cells With Productive IGK Contig** | 86.60% | 84.90% | 87.10% | 88.70% |
| **Cells With Productive IGL Contig** | 18.60% | 18.20% | 16.00% | 13.40% |

**Table S3. Reagents and resources identifier**

| REAGENT or RESOURCE | SOURCE | | IDENTIFIER |
| --- | --- | --- | --- |
| Antibodies |  | |  |
| LIVE/DEAD Aqua Dead Cell Stain | ThermoFisher | | Cat# L34966 |
| 573 mouse IgM Hybridoma | Leonard Evans, Rocky Mountain Laboratories (MTA); (*100*) | | N/A |
| 83A25 rat IgG2A Hybridoma | Leonard Evans, Rocky Mountain Laboratories (MTA); (*113*) | | N/A |
| mAb 34 mouse IgG2b | Gifted from W. Mothes, Yale University; (*25*) | | N/A |
| Rat IgG2a isotype | BD Pharmingen | | Cat# 553992; RRID: AB_395189 |
| Biotin mouse anti-rat IgG2a | BD Pharmingen | | Cat# 553894; RRID: AB_395122 |
| PE Streptavidin | BioLegend | | Cat# 405245 |
| Alexa Fluor™ 647—R-Phycoerythrin Streptavidin | Invitrogen | | Cat# S20992 |
| Goat anti-Mouse IgG (H+L), APC | Invitrogen | | Cat# A28181, RRID: AB_2536165 |
| F(ab')₂ Goat Anti-Mouse IgM, APC | Jackson | | Cat# 115-136-075; RRID: AB_2338650 |
| Goat Anti-Mouse IgG, Human ads-HRP | Southern Biotech | | Cat# 103005; RRID: AB_2619742 |
| Goat Anti-Mouse IgM, Human ads-HRP | Southern Biotech | | Cat# 1020-05; RRID: AB_2794201 |
| Goat Anti-Mouse Ig, Human ads-HRP | Southern Biotech | | Cat# 1010-05; RRID: AB_2728714 |
| Goat Anti-Mouse Ig, Human ads-UNLB | Southern Biotech | | Cat# 1010-01; RRID: AB_2794121 |
| Goat anti-Rat IgG (H+L) Secondary Antibody, HRP | ThermoFisher | | Cat# 31470; RRID: AB_228356 |
| His-Tag (D3I1O) XP® Rabbit mAb, HRP | Cell signaling | | Cat# 12688; RRID: AB_2797993 |
| Mouse IGHV1-53 mAb, γ2c | This study | | N/A |
| Mouse IGHV6-3 mAb, γ2c | This study | | N/A |
| Mouse IGHV7-3 mAb, γ2c | This study | | N/A |
| Mouse DS6-7_C1 mAb, γ2c | This study | | N/A |
| Mouse DS6-7_C2 mAb, γ2c | This study | | N/A |
| Mouse DS6-7_C3 mAb, γ2c | This study | | N/A |
| Mouse DS7-6_C1 mAb, γ2c | This study | | N/A |
| Mouse DS7-6_C2 mAb, γ2c | This study | | N/A |
| Mouse DS7-6_C3 mAb, γ2c | This study | | N/A |
| Mouse DS1-6_C1 mAb, γ2c | This study | | N/A |
| Mouse DS1-6_C2 mAb, γ2c | This study | | N/A |
| Mouse DS1-6_C3 mAb, γ2c | This study | | N/A |
| Mouse IGHV6-3 – human IgG1-RGY, γ1 | This study | | N/A |
| Human IGHV3-15 mAb, γ1 | This study | | N/A |
| Human IGHV1-8 mAb, γ1 | This study | | N/A |
| Mouse monoclonal anti-Lancefield Group A Carbohydrate (clone HGAC78, m) | Gifted by J. Kearney, University of Alabama at Birmingham; (*41*) | | N/A |
| Mouse monoclonal anti-Lancefield Group A Carbohydrate (clone HGAC39, g3) | Gifted by J. Kearney, University of Alabama at Birmingham; (*42*) | | N/A |
| Anti-mouse CD45, Alexa Fluor 488 | BioLegend | | Cat# 103122; RRID: AB_493531 |
| Anti-mouse CD3, Brilliant Violet 605™ | BioLegend | | Cat# 100237; RRID: AB_2562039 |
| Anti-mouse CD19, Brilliant Violet 421™ | BioLegend | | Cat# 115538; RRID: AB_11203527 |
| Anti-mouse CD5, FITC | BioLegend | | Cat# 100606; RRID: AB_312735 |
| Anti-mouse CD23, Brilliant Violet 510™ | BioLegend | | Cat# 101623; RRID: AB_2563705 |
| Anti-mouse CD21/35, APC/Cyanine7 | BioLegend | | Cat# 123417; RRID: AB_1953274 |
| Bacterial and Virus Strains |  | |  |
| One Shot™ TOP10 | ThermoFisher | | Cat# C404010 |
| ERV | (*7*) | | N/A |
| SARS-CoV-2 | BEI Resources | | NR-52281 |
| A/Puerto Rico/8/34 (H1N1) | Gifted by H. Hasegawa, National Institute of Infectious Diseases, Tokyo | | N/A |
| A/Udorn/307/72 (H3N2) | Gifted by R. Lamb, Northwestern University | | N/A |
| HSV-1 | Gifted by D. Knipe, Harvard Medical School | | N/A |
| HSV-2 | Gifted by D. Knipe, Harvard Medical School | | N/A |
| Chemicals, Peptides, and Recombinant Proteins | |  | |
| EZ-Link™ Sulfo-NHS-Biotin | ThermoFisher | | Cat# 21326 |
| Standard LPS | InvivoGen | | Cat# tlrl-eblps |
| R848 (Resiquimod) | InvivoGen | | Cat# tlrl-r848 |
| Pam3CSK | InvivoGen | | Cat# tlrl-pms |
| CpG | TriLink BioTechnologies; (*114*) | | N/A |
| Poly(I:C) | InvivoGen | | Cat# tlrl-picw |
| SLR14 | (*56*) | | N/A |
| Halt Protease Inhibitors ThermoScientific | ThermoFisher | | Cat# 78446 |
| TRIzol Reagent | Invitrogen | | Cat# 15596018 |
| ERV Envelope protein, His Tag | This study | | N/A |
| ERV Glycogag protein, His Tag | This study | | N/A |
| SARS-CoV-2 S protein, His Tag | Acro Biosystems | | Cat# SPN-C52H9 |
| HIV-1 gp120 Protein (group M, subtype CRF07_BC) (His Tag) | Sinobiological | | Cat# 11233-V08H |
| Influenza A A/Wisconsin/588/2019 (H1N1) Hemagglutinin (HA) Protein, His Tag | Acro Biosystems | | Cat# HA1-V52H3 |
| Influenza A H3N2 (A/X-31) Hemagglutinin / HA Protein (His Tag) | Sinobiological | | Cat# 40059-V08H |
| SARS S1 protein, His Tag | Acro Biosystems | | Cat# S1N-S52H5 |
| TNFRSF9, His Tag | Gifted by A. Ring, Yale University | | N/A |
| SCGB2A2, His Tag | Gifted by A. Ring, Yale University | | N/A |
| IL-1R, His Tag | Gifted by A. Ring, Yale University | | N/A |
| N-acetylglucosamine | Vector Lab | | Cat# S-9002 |
| α-methylmannoside | Vector Lab | | Cat# S-9005 |
| Critical Commercial Assays |  | |  |
| Ni-NTA Agarose | QIAGEN | | Cat# 30210 |
| Pierce™ Protein G Agarose | ThermoFisher | | Cat# 20397 |
| iTaq™ Universal SYBR® Green Supermix | Bio-Rad | | Cat# 1725124 |
| SuperSignal™ West Pico PLUS Chemiluminescent Substrate | ThermoFisher | | Cat# 34577 |
| Pierce™ ECL Western Blotting Substrate | ThermoFisher | | Cat# 32209 |
| eBioscience™ TMB Solution (1X) | Invitrogen | | Cat# 00-4201-56 |
| Shrimp Alkaline Phosphatase (rSAP) | ThermoFisher | | Cat# M0371S |
| T4 DNA Ligase | NEB | | Cat# M0202S |
| iScript™ cDNA Synthesis Kit | Bio-Rad | | Cat# 1708890 |
| SuperScript™ III CellsDirect™ cDNA Synthesis Kit | ThermoFisher | | Cat# 18080200 |
| Retro-X™ Concentrator | TAKARA | | Cat# 631456 |
| Lipofectamine™ 2000 Transfection Reagent | ThermoFisher | | Cat# 11668019 |
| Gibson Assembly® Master Mix | NEB | | Cat# E2611L |
| Biorad Precision plus protein ladder | Biorad | | Cat# 1610374 |
| EasySep™ Mouse CD4 T cell isolation kit | STEMCELL | | Cat# 19852 |
| EasySep™ Mouse Pan-B Cell Isolation Kit | STEMCELL | | Cat# 19844 |
| Expi293™ Expression Medium | ThermoFisher | | Cat# A1435101 |
| ExpiFectamine™ 293 Transfection Kit | ThermoFisher | | Cat# A14524 |
| Lipofectamine™ 2000 Transfection reagent | Invitrogen | | Cat# 11668019 |
| O-Glycosidase & Neuraminidase Bundle | NEB | | Cat# E0540S |
| PNGase F | NEB | | Cat# P0709S |
| β-N-Acetylglucosaminidase S | NEB | | Cat# P0744S |
| Q5® High-Fidelity 2X Master Mix | NEB | | Cat# M0492S |
| Lectin Kit III, Biotinylated | Vector Lab | | Cat# BK-3000 |
| VECTASTAIN® Elite ABC-HRP Kit, Peroxidase (Standard) | Vector Lab | | Cat# PK-6100 |
| Carbo-Free Blocking Solution (10x Concentrate) | Vector Lab | | Cat# SP-5040-125 |
| In-Fusion® Snap Assembly Master Mix | TAKARA | | Cat# 638948 |
| Gibson Assembly® Master Mix | NEB | | Cat# E2611L |
| Zymoclean Gel DNA Recovery Kit | ZYMO RESEARCH | | Cat# D4008 |
| DNA Clean & Concentrator™ | ZYMO RESEARCH | | Cat# D4005 |
| Plasmid Plus Midi Kit | QIAGEN | | Cat# 12945 |
| QIAprep Spin Miniprep Kit | QIAGEN | | Cat# 27104 |
| RNeasy Mini Kit | QIAGEN | | Cat# 74104 |
| QIAShredder | QIAGEN | | Cat# 79654 |
| Plasmid Plus Maxi Kit | QIAGEN | | Cat# 12963 |
| Amicon Ultra-15 Centrifugal Filter Unit, 10,000 MWCO | Millipore | | Cat# UFC901024 |
| Vivaspin^®^ 2 30,000 MWCO | GE Healthcare | | Cat# VS0291 |
| Poly-Prep® Chromatography Columns | Bio-Rad | | Cat# 7311550 |
| Econo-Pac® Chromatography Columns | Bio-Rad | | Cat# 7321010 |
| Experimental Models: Cell Lines |  | |  |
| 293T | ATCC | | CRL-3216; RRID: CVCL_0063 |
| Expi293F™ | Gibco | | Cat# A14527 |
| DFJ8 avian fibroblast | Gifted by W. Mothes, Yale University | | N/A |
| Oligonucleotides |  | |  |
| Primer: Spliced Emv2 Env Forward: 5’-CCAGGGACCACCGACCCACCGT-3’ | IDT (*115*) | | N/A |
| Primer: Spliced Emv2 Env Reverse: 5′-TAGTCGGTCCCGGTAGGCCTCG-3′ | IDT (*115*) | | N/A |
| Primer: GAPDH Forward: 5’-GAAGGTCGGTGTGAACGGA-3’ | IDT | | N/A |
| Primer: GAPDH Reverse: 5’-GTTAGTGGGGTCTCGCTCCT-3’ | IDT | | N/A |
| MLV_Pol Forward: 5’-  TGGCTGACTGAGGCTAGAAAAGAGACTGT-3’ | IDT (*116*) | | N/A |
| MLV_Pol Reverse: 5’-  GCTTTTTGGACAGGTAGGCCAC-3’ | IDT (*116*) | | N/A |
| Primer: FMLV-IF-1F: 5'-GGCCTCCCTCCTGACCTTAGACG-3' | IDT | | N/A |
| Primer: FMLV-IF-1R: 5'-TAAGGGGATTAGGAGGTCCCGCG-3' | IDT | | N/A |
| Primer: FMLV-IF-2FS: 5'- GGCTTTAGTCCTGACTCAACAATACC-3' | IDT | | N/A |
| Primer: FMLV-IF-2FM: 5'-CGCGGGACCTCCTAATCCCCTTACCAGCTAAAACCACTAGAATACGAGCC-3' | IDT | | N/A |
| Primer: FMLV-IF-2R: 5'-TTTCCATAGGCTCCGCCCCC-3' | IDT | | N/A |
| Primer: FMLV-IF-3F: 5'-GGGGCGGAGCCTATGGAAAAACG-3' | IDT | | N/A |
| Primer: FMLV-IF-3R: 5'-CGTCTAAGGTCAGGAGGGAGGCC-3' | IDT | | N/A |
| Recombinant DNA |  | |  |
| Plasmid: pRVL-2 | Addgene | | Cat# 104580 |
| Plasmid: pRVL-1 | Addgene | | Cat# 104579 |
| Plasmid: FMLV-DEnvDGlycogag | This study | | N/A |
| Plasmid: pEZT_MLV_Env_SU_M | This study | | N/A |
| Plasmid: pEZT_MLV_Env_SU_V | This study | | N/A |
| Plasmid: pUC19-ERV | (*7*) | | N/A |
| Plasmid: pRVL-2_IGHV1-53 | This study | | N/A |
| Plasmid: pRVL-2_IGHV6-3 | This study | | N/A |
| Plasmid: pRVL-2_IGHV7-3 | This study | | N/A |
| Plasmid: pRVL-1_IGKV4 | This study | | N/A |
| Plasmid: pRVL-1_IGKV14 | This study | | N/A |
| Plasmid: pRVL-1_IGLV1 | This study | | N/A |
| Plasmid: pRVL-2_DS6-7_C1 | This study | | N/A |
| Plasmid: pRVL-2_DS6-7_C2 | This study | | N/A |
| Plasmid: pRVL-2_DS6-7_C3 | This study | | N/A |
| Plasmid: pRVL-2_DS7-6_C1 | This study | | N/A |
| Plasmid: pRVL-2_DS7-6_C2 | This study | | N/A |
| Plasmid: pRVL-2_DS7-6_C3 | This study | | N/A |
| Plasmid: pRVL-2_DS1-6_C1 | This study | | N/A |
| Plasmid: pRVL-2_DS1-6_C2 | This study | | N/A |
| Plasmid: pRVL-2_DS1-6_C3 | This study | | N/A |
| Software and Algorithms |  | |  |
| FlowJo | TreeStar Inc | | <https://www.flowjo.com/> |
| GraphPad Prism 9 | GraphPad Software | | <https://www.graphpad.com/> |
| Cell Ranger | 10X Genomics | | <https://support.10xgenomics.com/single-cell-gene-expression/software/overview/welcome> |
| Change-O | (*105*) | | <https://changeo.readthedocs.io/en/stable/> |
| Alakazam | (*105*) | | <https://alakazam.readthedocs.io/en/stable/> |
| SHazaM | (*105*) | | https://shazam.readthedocs.io/en/stable/ |
| Treemap | Martijn Tennekes | | [https://CRAN.R-project.org/package=treemap](https://cran.r-project.org/package=treemap) |
| Seurat V3 | (*103*) | | <https://satijalab.org/seurat/index.html> |
| ScRepertoire | (*108*) | | <https://ncborcherding.github.io/vignettes/vignette.html> |
| Enrichr | (*104*) | | <https://cran.r-project.org/web/packages/enrichR/index.html> |
| TMHMM - 2.0 | DTU Health Tech | | <https://services.healthtech.dtu.dk/service.php?TMHMM-2.0> |
| IgBLAST | (*106*) | | https://bio.tools/igblast |
| msa | (*117*) | | <https://bioconductor.org/packages/release/bioc/html/msa.html> |
| IMGT/High-Vquest | (*107*) | | [https://www.imgt.org](https://www.imgt.org/) |
